# Supplementary material for: The Evolution and Characterization of the RNA Interference Pathways in Lophotrochozoa
Source: Genome Biol Evol. 2024 May 7;16(5):evae098. doi: 10.1093/gbe/evae098 (PMC11114477; doi:10.1093/gbe/evae098)
Supplement: evae098_Supplementary_Data [file evae098_supplementary_data.zip › Supplementary_materials.zip!/selected_miRNAs/NoGe_selected_miRNAs/mirdeep_pdf_files/NMRB01000662.1_Notospermus_geniculatus_strain_Ushimado_scaffold662,_whole_genome_shotgun_sequence_18050.pdf]

Provisional ID	:	NMRB01000662.1_Notospermus_geniculatus_strain_Ushimado_scaffold662,_whole_genome_shotgun_sequence_18050
Score total	:	245011.5
Score for star read(s)	:	3.9
Score for read counts	:	245002.7
Score for mfe	:	1.9
Score for randfold	:	0
Score for cons. seed	:	3
Total read count	:	480574
Mature read count	:	471927
Loop read count	:	16
Star read count	:	8631

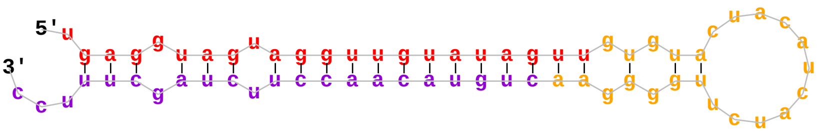

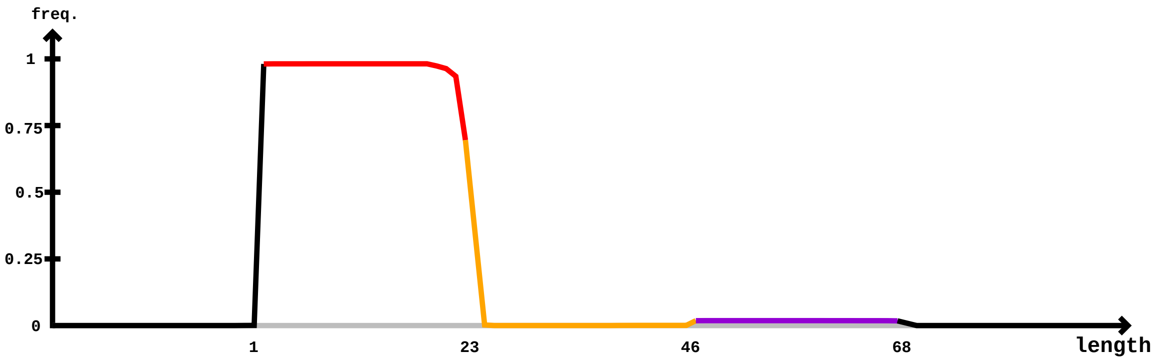

Mature Star

```
5'- caggagugggccccucagucugagguaguagguuguauaguuguguacuacaucaucuuggggaacuguacaaccuucuagcuuucccagagggcgccucaguacaacau  -3'  obs
    caggagugggccccucagucugagguaguagguuguauaguuguguacuacaucaucuuggggaacuguacaaccuucuagcuuucccagagggcgccucaguacaacau       exp
    ....(.((((((((((.....(((.(((.(((((((((((((.(.((...........)).).))))))))))))).))).))).....))))).))).)).).......   reads  mm  sample
    ..................ucugagguaguagguuguauag......................................................................   1      0   Mm3
    ..................ucugagguaguagguuguauagu.....................................................................   4      0   Mm3
    ..................ucugagguaguagguuguauaguu....................................................................   3      0   Mm3
    ...................cugagguaguagguuguauagu.....................................................................   2      0   Mm3
    ...................cugagguaguagguuguauaguu....................................................................   5      0   Mm3
    ...................cugagguaguagguuguauaguuU...................................................................   1      1   Mm3
    ....................ugagguaguagguuguau........................................................................   146    0   Mm3
    ....................ugGgguaguagguuguau........................................................................   1      1   Mm3
    ....................ugagguaguaggAuguau........................................................................   1      1   Mm3
    ....................uAagguaguagguuguau........................................................................   1      1   Mm3
    ....................ugagguaguagguuguUu........................................................................   9      1   Mm3
    ....................ugagguaguagguuguaA........................................................................   7      1   Mm3
    ....................ugUgguaguagguuguau........................................................................   1      1   Mm3
    ....................ugagguaguagguuguaC........................................................................   2      1   Mm3
    ....................ugagguaguagguuguaG........................................................................   3      1   Mm3
    ....................ugagguaguaUguuguaua.......................................................................   1      1   Mm3
    ....................ugagguaguagguuguUua.......................................................................   2      1   Mm3
    ....................ugagguaguagguuguauU.......................................................................   126    1   Mm3
    ....................ugagguaguagguuguaAa.......................................................................   13     1   Mm3
    ....................ugagguaguagguuguaua.......................................................................   125    0   Mm3
    ....................ugagguaguagguuguaGa.......................................................................   2      1   Mm3
    ....................ugagguaguaCguuguauag......................................................................   1      1   Mm3
    ....................ugGgguaguagguuguauag......................................................................   1      1   Mm3
    ....................ugagguaguagguuguaCag......................................................................   1      1   Mm3
    ....................ugagguaguagguuguauaA......................................................................   10     1   Mm3
    ....................uCagguaguagguuguauag......................................................................   1      1   Mm3
    ....................Ngagguaguagguuguauag......................................................................   1      1   Mm3
    ....................ugagguaguagguuguaGag......................................................................   2      1   Mm3
    ....................ugagguaguagguuguauag......................................................................   543    0   Mm3
    ....................uUagguaguagguuguauag......................................................................   1      1   Mm3
    ....................ugagguaguagguuguUuag......................................................................   1      1   Mm3
    ....................ugagguaguGgguuguauag......................................................................   1      1   Mm3
    ....................ugUgguaguagguuguauag......................................................................   1      1   Mm3
```

Mature   Star

```
caggagugggccccucagucugagguaguagguuguauaguuguguacuacaucaucuuggggaacuguacaaccuucuagcuuucccagagggcgccucaguacaacau
....................ugagguaguagguuguaAag......................................................................  2      1  Mm3
....................uAagguaguagguuguauag......................................................................  1      1  Mm3
....................ugagguaguagguuguauaU......................................................................  19     1  Mm3
....................ugagguaguagguuguauGgu.....................................................................  1      1  Mm3
....................Ngagguaguagguuguauagu.....................................................................  2      1  Mm3
....................ugaUguaguagguuguauagu.....................................................................  1      1  Mm3
....................ugagguaguaUguuguauagu.....................................................................  8      1  Mm3
....................ugagguaguagguuguaCagu.....................................................................  38     1  Mm3
....................ugagguaguGgguuguauagu.....................................................................  2      1  Mm3
....................uNagguaguagguuguauagu.....................................................................  1      1  Mm3
....................ugagguaguagguuguauagA.....................................................................  20     1  Mm3
....................ugagguagCagguuguauagu.....................................................................  1      1  Mm3
....................ugagguaguagguGguauagu.....................................................................  1      1  Mm3
....................ugagguaguagguuguGuagu.....................................................................  8      1  Mm3
....................uAagguaguagguuguauagu.....................................................................  3      1  Mm3
....................ugagguaguaCguuguauagu.....................................................................  4      1  Mm3
....................Ggagguaguagguuguauagu.....................................................................  2      1  Mm3
....................ugagguaguagguuguauagu.....................................................................  6423   0  Mm3
....................Cgagguaguagguuguauagu.....................................................................  1      1  Mm3
....................ugagAuaguagguuguauagu.....................................................................  1      1  Mm3
....................ugagguaguaAguuguauagu.....................................................................  11     1  Mm3
....................ugagguaAuagguuguauagu.....................................................................  1      1  Mm3
....................ugagguagGagguuguauagu.....................................................................  1      1  Mm3
....................ugGgguaguagguuguauagu.....................................................................  7      1  Mm3
....................uUagguaguagguuguauagu.....................................................................  2      1  Mm3
....................ugaAguaguagguuguauagu.....................................................................  1      1  Mm3
....................ugagguaguagguCguauagu.....................................................................  1      1  Mm3
....................ugagguUguagguuguauagu.....................................................................  3      1  Mm3
....................ugagguaguaggCuguauagu.....................................................................  1      1  Mm3
....................ugagguaguagguuguaAagu.....................................................................  25     1  Mm3
....................ugagguaguagguuguaGagu.....................................................................  29     1  Mm3
....................ugagguaguagguuguauaUu.....................................................................  2      1  Mm3
....................Agagguaguagguuguauagu.....................................................................  3      1  Mm3
....................ugagguaguagguugGauagu.....................................................................  1      1  Mm3
....................ugagguaguagguuguauUgu.....................................................................  3      1  Mm3
....................ugagguaguUgguuguauagu.....................................................................  3      1  Mm3
....................ugagguaUuagguuguauagu.....................................................................  1      1  Mm3
....................ugagguaguagguuguauagG.....................................................................  5      1  Mm3
....................ugagguaguagguuguUuagu.....................................................................  7      1  Mm3
....................ugagguaguagguuguauagC.....................................................................  5      1  Mm3
....................ugagguaguagguuguCuagu.....................................................................  25     1  Mm3
....................ugagguaguagguAguauagu.....................................................................  2      1  Mm3
....................ugUgguaguagguuguauagu.....................................................................  5      1  Mm3
....................ugagguGguagguuguauagu.....................................................................  3      1  Mm3
....................ugagguaguagguuguauaAu.....................................................................  2      1  Mm3
....................uUagguaguagguuguauaguu....................................................................  1      1  Mm3
....................ugagguaguagUuuguauaguu....................................................................  1      1  Mm3
....................ugagguaguagguuguauCguu....................................................................  4      1  Mm3
....................ugCgguaguagguuguauaguu....................................................................  15     1  Mm3
....................ugagguaguagguuguaCaguu....................................................................  87     1  Mm3
....................ugagguaguaUguuguauaguu....................................................................  13     1  Mm3
....................ugagguGguagguuguauaguu....................................................................  1      1  Mm3
....................ugagguaguagguuguauaguu....................................................................  14796  0  Mm3
....................ugaggNaguagguuguauaguu....................................................................  1      1  Mm3
....................ugagguaguagguGguauaguu....................................................................  2      1  Mm3
....................ugagguaguagguuguauaguA....................................................................  115    1  Mm3
....................ugagguaguagguuguauaguC....................................................................  4      1  Mm3
....................ugagguagCagguuguauaguu....................................................................  2      1  Mm3
....................ugagguaguagguuguauGguu....................................................................  2      1  Mm3
....................ugagguaguagguAguauaguu....................................................................  1      1  Mm3
....................ugagguaguagguuguauaCuu....................................................................  1      1  Mm3
....................ugagguaguaggGuguauaguu....................................................................  2      1  Mm3
....................ugaggAaguagguuguauaguu....................................................................  5      1  Mm3
....................ugagguaguagguuAuauaguu....................................................................  1      1  Mm3
....................ugagguaguagguuguauaUuu....................................................................  2      1  Mm3
....................ugaggCaguagguuguauaguu....................................................................  3      1  Mm3
....................ugagguaguagguuguUuaguu....................................................................  18     1  Mm3
....................ugagUuaguagguuguauaguu....................................................................  2      1  Mm3
....................ugagguaguagguuguauaAuu....................................................................  1      1  Mm3
....................ugagguaguagguuguauUguu....................................................................  4      1  Mm3
```

```
                    Mature                                       Star
caggagugggccccucagucugagguaguagguuguauaguuguguacuacaucaucuuggggaacuguacaaccuucuagcuuucccagagggcgccucaguacaacau
....................ugaAguaguagguuguauaguu....................................................................    1          1          Mm3
....................ugUgguaguagguuguauaguu....................................................................    14         1          Mm3
....................ugagguaguagguuguCuaguu....................................................................    39         1          Mm3
....................ugagguaguagguuguaAaguu....................................................................    62         1          Mm3
....................ugagguaUuagguuguauaguu....................................................................    1          1          Mm3
....................ugagguaguagguuCuauaguu....................................................................    1          1          Mm3
....................ugagguaguagguugGauaguu....................................................................    1          1          Mm3
....................ugagguaguagguCguauaguu....................................................................    2          1          Mm3
....................ugagguaguagguuguaGaguu....................................................................    75         1          Mm3
....................ugagguaguaAguuguauaguu....................................................................    27         1          Mm3
....................ugGgguaguagguuguauaguu....................................................................    29         1          Mm3
....................ugaCguaguagguuguauaguu....................................................................    1          1          Mm3
....................ugagguaguagguuguauaguG....................................................................    13         1          Mm3
....................ugagguagAagguuguauaguu....................................................................    2          1          Mm3
....................ugagguaguCgguuguauaguu....................................................................    2          1          Mm3
....................ugagguCguagguuguauaguu....................................................................    1          1          Mm3
....................ugagguaguUgguuguauaguu....................................................................    5          1          Mm3
....................Agagguaguagguuguauaguu....................................................................    3          1          Mm3
....................uAagguaguagguuguauaguu....................................................................    8          1          Mm3
....................ugagguagGagguuguauaguu....................................................................    2          1          Mm3
....................ugagguUguagguuguauaguu....................................................................    3          1          Mm3
....................Cgagguaguagguuguauaguu....................................................................    7          1          Mm3
....................Ggagguaguagguuguauaguu....................................................................    1          1          Mm3
....................ugagguaguaCguuguauaguu....................................................................    10         1          Mm3
....................ugagguaguagguuUuauaguu....................................................................    1          1          Mm3
....................ugagguaguGgguuguauaguu....................................................................    12         1          Mm3
....................ugagguaguagguuguauagCu....................................................................    3          1          Mm3
....................ugagguaguaggCuguauaguu....................................................................    1          1          Mm3
....................ugaggGaguagguuguauaguu....................................................................    1          1          Mm3
....................Ngagguaguagguuguauaguu....................................................................    5          1          Mm3
....................ugagguaCuagguuguauaguu....................................................................    3          1          Mm3
....................ugagguaguagguuguGuaguu....................................................................    13         1          Mm3
....................uCagguaguagguuguauaguu....................................................................    1          1          Mm3
....................ugagguaguagguuguauagAu....................................................................    1          1          Mm3
....................ugagguNguagguuguauaguu....................................................................    1          1          Mm3
....................ugagguaguagguuguauaguAg...................................................................    4          1          Mm3
....................ugagguaguagguuguauaguug...................................................................    23         0          Mm3
....................ugagguaguagguuguauaguuU...................................................................    2816       1          Mm3
....................ugagguaguagguuguauaguuA...................................................................    268        1          Mm3
....................Ngagguaguagguuguauaguug...................................................................    1          1          Mm3
....................ugagguaguagguuguauaguGg...................................................................    1          1          Mm3
....................Agagguaguagguuguauaguug...................................................................    1          1          Mm3
....................ugagguaguagguuguCuaguug...................................................................    1          1          Mm3
....................ugagguaguagguuguauaguuC...................................................................    13         1          Mm3
....................ugagguaguaCguuguauaguug...................................................................    1          1          Mm3
....................ugagguaguagguuguauaguuAu..................................................................    22         1          Mm3
....................ugagguaguagguuguauaguuCu..................................................................    1          1          Mm3
....................ugagguaguagguuguauaguAgu..................................................................    1          1          Mm3
....................ugagguaguagguuguauaguuUu..................................................................    94         1          Mm3
....................ugagguaguagguuguauaguugugG................................................................    1          1          Mm3
....................ugagguaguagguuguauaguugugu................................................................    1          0          Mm3
....................ugagguaguagguuguaGaguuguguacu.............................................................    1          1          Mm3
.....................gagguaguagguuguauagu.....................................................................    3          0          Mm3
.....................Uagguaguagguuguauaguu....................................................................    1          1          Mm3
.....................gagguaguagguuguauaguu....................................................................    10         0          Mm3
.....................gagguaguagguuguauaguuU...................................................................    2          1          Mm3
.....................gagguaguagguuguauaguuA...................................................................    1          1          Mm3
......................agguaguagguuguauaguu....................................................................    1          0          Mm3
......................agguaguagguuguauaguug...................................................................    1          0          Mm3
......................agguaguagguuguauaguuU...................................................................    2          1          Mm3
.......................gguaguagguuguauagu.....................................................................    1          0          Mm3
.......................gguaguagguuguauaguuU...................................................................    1          1          Mm3
.........................................uguguacuacaucaucuugggU...............................................    1          1          Mm3
..........................................guguacuacaucaucuugggg...............................................    1          0          Mm3
..........................................guguacuacaucaucuuggggaa.............................................    1          0          Mm3
................................................uacaucaucuuggggaacuguacaacc...................................    2          0          Mm3
..................................................caucaucuuggggaacuguacaaccuuc................................    1          0          Mm3
......................................................aucuuggggaacuguacaaccuucuagc............................    1          0          Mm3
......................................................aucuuggggaacuguacaaccuucuagcuuucc.......................    4          0          Mm3
.......................................................ucuuggggaacuguacaaccuucuagU............................    1          1          Mm3
```

Mature			
caggagugggccccucagucugagguaguagguuguauaguuguguacuacaucaucuuggggaacuguacaaccuucuagcuuucccagagggcgccucaguacaacau			
....uuggggaacuguacaaccuucu...............................	1	0	Mm3
....ggggaacuguacaaccuucuagcuuucc.......................	2	0	Mm3
....gggaacuguacaaccuucuag.............................	2	0	Mm3
....gggaacuguacaaccuucuagc............................	1	0	Mm3
....aacuguacaaccuucuagcuuucc.......................	1	0	Mm3
....cuguacaaccuucuagcC...........................	1	1	Mm3
....cuguacaaccuucuagcu...........................	3	0	Mm3
....cuguacaaccuucuagcuuu.........................	2	0	Mm3
....cuguacaaccuucuagcuuuc........................	30	0	Mm3
....cuguaUaaccuucuagcuuucc.......................	1	1	Mm3
....Uuguacaaccuucuagcuuucc.......................	4	1	Mm3
....cuUuacaaccuucuagcuuucc.......................	1	1	Mm3
....cuguacaaccuuUuagcuuucc.......................	2	1	Mm3
....cuguacaGccuucuagcuuucc.......................	1	1	Mm3
....cuguacaaccuucuagcuuucU.......................	10	1	Mm3
....cuguacaaccuucuagcGuucc.......................	6	1	Mm3
....cCguacaaccuucuagcuuucc.......................	1	1	Mm3
....cuguacaaccuucuagcAuucc.......................	9	1	Mm3
....Auguacaaccuucuagcuuucc.......................	1	1	Mm3
....cuguacaaccuucuagcuuucc.......................	1116	0	Mm3
....cuguacaaccuucuagUuuucc.......................	2	1	Mm3
....cuguacaaccuucuagcCuucc.......................	3	1	Mm3
....cuguacaaccuucuagGuuucc.......................	1	1	Mm3
....cuguacaaccGucuagcuuucc.......................	1	1	Mm3
....cuguacaaccuucuagAuuucc.......................	9	1	Mm3
....cuguacaaccAucuagcuuucc.......................	1	1	Mm3
....cuguacaaccCucuagcuuucc.......................	1	1	Mm3
....cuguacaaccuucuagcuuuccA......................	48	1	Mm3
....cuguacaaccuucuagcuuuccU......................	32	1	Mm3
....uguacaaccuucuagcuuucc.......................	8	0	Mm3
....uguacaaccuucuagcuuuccU......................	4	1	Mm3
....uguacaaccuucuagcuuuccG......................	1	1	Mm3
....uguacaaccuucuagcuuuccA......................	3	1	Mm3
..................ucugagguaguagguuguauag..	4	0	Fm3
..................ucugagguaguagguuguauagu...	12	0	Fm3
...................cugagguaguagguuguauagu...	3	0	Fm3
...................cugagguaguagguuguauaguu..	11	0	Fm3
...................cugagguagCagguuguauaguu..	1	1	Fm3
...................cugagguaguGgguuguauaguu..	1	1	Fm3
....................ugagguaguagguuguaC..	23	1	Fm3
....................ugagguaguagCuuguau..	2	1	Fm3
....................ugagguaguagguuguUu..	96	1	Fm3
....................ugagguaguCgguuguau..	1	1	Fm3
....................ugagguaguagguAguau..	2	1	Fm3
....................ugagguaguagguuguaA..	134	1	Fm3
....................ugagguaguagguuguau..	2865	0	Fm3
....................ugagguUguagguuguau..	1	1	Fm3
....................Agagguaguagguuguau..	1	1	Fm3
....................ugagguaguaAguuguau..	4	1	Fm3
....................ugagguaguaggAuguau..	1	1	Fm3
....................ugagguaAuagguuguau..	1	1	Fm3
....................ugagguaguagguuguCu..	6	1	Fm3
....................Cgagguaguagguuguau..	1	1	Fm3
....................ugagguaguaUguuguau..	6	1	Fm3
....................ugagguaguagguuguaG..	8	1	Fm3
....................ugagCuaguagguuguau..	1	1	Fm3
....................ugagguaguagguuguGu..	5	1	Fm3
....................ugagguaguGgguuguau..	1	1	Fm3
....................ugGgguaguagguuguau..	6	1	Fm3
....................ugagguaguagguuguauU...	1945	1	Fm3
....................ugaggGaguagguuguaua...	1	1	Fm3
....................ugagguaguagguuguaGa...	18	1	Fm3
....................ugagguaguaUguuguaua...	3	1	Fm3
....................ugagguaguGgguuguaua...	1	1	Fm3
....................ugagguaguaCguuguaua...	1	1	Fm3
....................ugagguaguUgguuguaua...	1	1	Fm3
....................ugagguCguagguuguaua...	1	1	Fm3
....................ugagguaguagguuguGua...	2	1	Fm3
....................ugagguaguagguuguauC...	1	1	Fm3

Mature **Star**

```
caggagugggccccucagucugagguaguagguuguauaguuguguacuacaucaucuuggggaacuguacaaccuucuagcuuucccagagggcgccucaguacaacau
....................ugagguaguagguuguaCa.......................................................................   11      1   Fm3
....................Agagguaguagguuguaua.......................................................................   2       1   Fm3
....................ugagguaguagguuguaAa.......................................................................   106     1   Fm3
....................Cgagguaguagguuguaua.......................................................................   1       1   Fm3
....................ugGgguaguagguuguaua.......................................................................   1       1   Fm3
....................ugagguaguaAguuguaua.......................................................................   2       1   Fm3
....................ugagguaguagguuguUua.......................................................................   1       1   Fm3
....................ugagguaguagguuAuaua.......................................................................   3       1   Fm3
....................ugCgguaguagguuguaua.......................................................................   3       1   Fm3
....................ugagguaguagguuguaua.......................................................................   1441    0   Fm3
....................ugagguaguagguCguaua.......................................................................   1       1   Fm3
....................ugagguaguagguuguaCag......................................................................   51      1   Fm3
....................uCagguaguagguuguauag......................................................................   1       1   Fm3
....................uAagguaguagguuguauag......................................................................   4       1   Fm3
....................ugagguaguagguuguaGag......................................................................   56      1   Fm3
....................ugaggCaguagguuguauag......................................................................   2       1   Fm3
....................Cgagguaguagguuguauag......................................................................   4       1   Fm3
....................ugUgguaguagguuguauag......................................................................   7       1   Fm3
....................Ngagguaguagguuguauag......................................................................   1       1   Fm3
....................uUagguaguagguuguauag......................................................................   3       1   Fm3
....................ugagguaguagguuguauUg......................................................................   1       1   Fm3
....................ugagguaguagguuguCuag......................................................................   11      1   Fm3
....................ugagguaguagguuguaAag......................................................................   46      1   Fm3
....................ugagguUguagguuguauag......................................................................   3       1   Fm3
....................ugagguagAagguuguauag......................................................................   1       1   Fm3
....................ugagguaguagguuguauGg......................................................................   1       1   Fm3
....................ugagguaguagguuguauaA......................................................................   147     1   Fm3
....................ugagCuaguagguuguauag......................................................................   1       1   Fm3
....................ugagguaguagguuguGuag......................................................................   10      1   Fm3
....................ugagguaguaCguuguauag......................................................................   9       1   Fm3
....................Agagguaguagguuguauag......................................................................   3       1   Fm3
....................ugagguaguagguugAauag......................................................................   1       1   Fm3
....................ugagguaguagguuguUuag......................................................................   11      1   Fm3
....................ugagguaguagguuguauaU......................................................................   177     1   Fm3
....................ugagguaguagguuUuauag......................................................................   1       1   Fm3
....................ugagguagCagguuguauag......................................................................   4       1   Fm3
....................ugagguaguagguuguauCg......................................................................   1       1   Fm3
....................ugagguaUuagguuguauag......................................................................   1       1   Fm3
....................ugagguaguagguuguauag......................................................................   9248    0   Fm3
....................ugagguaguagguCguauag......................................................................   6       1   Fm3
....................ugCgguaguagguuguauag......................................................................   6       1   Fm3
....................ugagguaguaUguuguauag......................................................................   9       1   Fm3
....................ugagguaguGgguuguauag......................................................................   5       1   Fm3
....................ugagguaguagguGguauag......................................................................   1       1   Fm3
....................ugagguaguaggCuguauag......................................................................   1       1   Fm3
....................ugagguNguagguuguauag......................................................................   1       1   Fm3
....................ugGgguaguagguuguauag......................................................................   14      1   Fm3
....................ugaggGaguagguuguauag......................................................................   3       1   Fm3
....................ugaggNaguagguuguauag......................................................................   2       1   Fm3
....................ugagguaguagguugCauag......................................................................   2       1   Fm3
....................ugagguaguagguAguauag......................................................................   2       1   Fm3
....................ugagguaguaAguuguauag......................................................................   15      1   Fm3
....................ugaggAaguagguuguauag......................................................................   2       1   Fm3
....................ugagguGguagguuguauag......................................................................   2       1   Fm3
....................ugagguaguagguuCuauagu.....................................................................   4       1   Fm3
....................ugagguagCagguuguauagu.....................................................................   6       1   Fm3
....................ugagguaguaUguuguauagu.....................................................................   55      1   Fm3
....................Agagguaguagguuguauagu.....................................................................   17      1   Fm3
....................ugagguaguagguuUuauagu.....................................................................   4       1   Fm3
....................uCagguaguagguuguauagu.....................................................................   6       1   Fm3
....................ugagguaguagguuguauagG.....................................................................   14      1   Fm3
....................ugagguaguagguuguauagu.....................................................................   63418   0   Fm3
....................ugagguaguagguuguCuagu.....................................................................   131     1   Fm3
....................ugagguaguagAuuguauagu.....................................................................   2       1   Fm3
....................Cgagguaguagguuguauagu.....................................................................   17      1   Fm3
....................ugagguaguagguugCauagu.....................................................................   5       1   Fm3
....................ugCgguaguagguuguauagu.....................................................................   37      1   Fm3
....................ugagguaguagguuguaCagu.....................................................................   404     1   Fm3
....................ugagAuaguagguuguauagu.....................................................................   3       1   Fm3
....................uUagguaguagguuguauagu.....................................................................   22      1   Fm3
```

```
                    Mature                                       Star
caggagugggccccucagucugagguaguagguuguauaguuguguacuacaucaucuuggggaacuguacaaccuucuagcuuucccagagggcgccucaguacaacau
....................ugagguaguagguugGauagu.....................................................................   1       1   Fm3
....................ugaCguaguagguuguauagu.....................................................................   8       1   Fm3
....................uAagguaguagguuguauagu.....................................................................   37      1   Fm3
....................ugaggCaguagguuguauagu.....................................................................   14      1   Fm3
....................ugNgguaguagguuguauagu.....................................................................   1       1   Fm3
....................ugagguaguagguAguauagu.....................................................................   12      1   Fm3
....................ugagguaguagCuuguauagu.....................................................................   2       1   Fm3
....................ugagguaguagguGguauagu.....................................................................   6       1   Fm3
....................ugagguaguagguuguauagA.....................................................................   440     1   Fm3
....................ugGgguaguagguuguauagu.....................................................................   67      1   Fm3
....................ugagCuaguagguuguauagu.....................................................................   1       1   Fm3
....................ugagguaguagguuguaGagu.....................................................................   363     1   Fm3
....................ugagguaguagguuguaAagu.....................................................................   273     1   Fm3
....................ugagguaguagguuguauGgu.....................................................................   3       1   Fm3
....................Ngagguaguagguuguauagu.....................................................................   27      1   Fm3
....................ugagguaUuagguuguauagu.....................................................................   10      1   Fm3
....................ugagguaCuagguuguauagu.....................................................................   2       1   Fm3
....................ugagguaguagUuuguauagu.....................................................................   2       1   Fm3
....................ugagguaguagguugAauagu.....................................................................   7       1   Fm3
....................ugagguCguagguuguauagu.....................................................................   4       1   Fm3
....................ugagguaguagguuguauagC.....................................................................   9       1   Fm3
....................ugagguaguagguuguauaCu.....................................................................   1       1   Fm3
....................ugagguagGagguuguauagu.....................................................................   5       1   Fm3
....................ugagguaguUgguuguauagu.....................................................................   8       1   Fm3
....................ugagguaguaAguuguauagu.....................................................................   98      1   Fm3
....................ugagguNguagguuguauagu.....................................................................   1       1   Fm3
....................Ggagguaguagguuguauagu.....................................................................   6       1   Fm3
....................ugagguaguagguuguUuagu.....................................................................   68      1   Fm3
....................ugagguaguCgguuguauagu.....................................................................   2       1   Fm3
....................ugaggGaguagguuguauagu.....................................................................   9       1   Fm3
....................ugagguUguagguuguauagu.....................................................................   23      1   Fm3
....................ugagguaguagguuguauaAu.....................................................................   14      1   Fm3
....................ugagguaAuagguuguauagu.....................................................................   1       1   Fm3
....................ugagguaguagguCguauagu.....................................................................   10      1   Fm3
....................ugUgguaguagguuguauagu.....................................................................   42      1   Fm3
....................ugagguaguaggCuguauagu.....................................................................   20      1   Fm3
....................ugagguaguagguuguauUgu.....................................................................   13      1   Fm3
....................ugaggAaguagguuguauagu.....................................................................   10      1   Fm3
....................ugaUguaguagguuguauagu.....................................................................   8       1   Fm3
....................ugagguaguagguuguGuagu.....................................................................   61      1   Fm3
....................ugaAguaguagguuguauagu.....................................................................   7       1   Fm3
....................ugagguagAagguuguauagu.....................................................................   8       1   Fm3
....................ugagguaguagguuguauaUu.....................................................................   34      1   Fm3
....................ugagguaguaCguuguauagu.....................................................................   48      1   Fm3
....................ugagguaguagguuguauCgu.....................................................................   4       1   Fm3
....................ugagguaguGgguuguauagu.....................................................................   14      1   Fm3
....................ugagguaguaggAuguauagu.....................................................................   5       1   Fm3
....................ugagguGguagguuguauagu.....................................................................   9       1   Fm3
....................ugagguaguagguuAuauagu.....................................................................   4       1   Fm3
....................ugaggNaguagguuguauagu.....................................................................   12      1   Fm3
....................ugagguaguagguuguaGaguu....................................................................   789     1   Fm3
....................ugagguaguagAuuguauaguu....................................................................   9       1   Fm3
....................ugagguaguagUuuguauaguu....................................................................   7       1   Fm3
....................ugaggNaguagguuguauaguu....................................................................   16      1   Fm3
....................ugagguaguagguuguauagAu....................................................................   4       1   Fm3
....................ugagguaguGgguuguauaguu....................................................................   56      1   Fm3
....................ugagguaguagguuguauaguC....................................................................   16      1   Fm3
....................ugagguagAagguuguauaguu....................................................................   19      1   Fm3
....................ugagguaguagguuUuauaguu....................................................................   4       1   Fm3
....................ugagguaguagguuguauaguu....................................................................   140887  0   Fm3
....................ugagguaguagguuguGuaguu....................................................................   136     1   Fm3
....................ugCgguaguagguuguauaguu....................................................................   104     1   Fm3
....................ugagguagGagguuguauaguu....................................................................   8       1   Fm3
....................ugagCuaguagguuguauaguu....................................................................   10      1   Fm3
....................ugagguaguagguuguauaCuu....................................................................   5       1   Fm3
....................ugagguaguagguAguauaguu....................................................................   27      1   Fm3
....................Agagguaguagguuguauaguu....................................................................   37      1   Fm3
....................uAagguaguagguuguauaguu....................................................................   63      1   Fm3
....................ugagguaCuagguuguauaguu....................................................................   3       1   Fm3
....................Ngagguaguagguuguauaguu....................................................................   80      1   Fm3
```

```
                    Mature                                       Star
caggagugggccccucagucugagguaguagguuguauaguuguguacuacaucaucuuggggaacuguacaaccuucuagcuuucccagagggcgccucaguacaacau
....................ugagAuaguagguuguauaguu....................................................................   15      1   Fm3
....................ugagguaguaggGuguauaguu....................................................................   2       1   Fm3
....................ugagguaguagguugCauaguu....................................................................   22      1   Fm3
....................ugagguaguagguuguauaguA....................................................................   687     1   Fm3
....................Cgagguaguagguuguauaguu....................................................................   58      1   Fm3
....................ugagguNguagguuguauaguu....................................................................   5       1   Fm3
....................ugagguaguagguuguCuaguu....................................................................   331     1   Fm3
....................ugagguaguaCguuguauaguu....................................................................   132     1   Fm3
....................ugagguaguagguuguaCaguu....................................................................   850     1   Fm3
....................ugagguaAuagguuguauaguu....................................................................   8       1   Fm3
....................ugaggGaguagguuguauaguu....................................................................   12      1   Fm3
....................ugagguGguagguuguauaguu....................................................................   42      1   Fm3
....................ugagguaguagguuguauaAuu....................................................................   5       1   Fm3
....................uNagguaguagguuguauaguu....................................................................   1       1   Fm3
....................ugagguaguUgguuguauaguu....................................................................   35      1   Fm3
....................ugGgguaguagguuguauaguu....................................................................   185     1   Fm3
....................ugagguUguagguuguauaguu....................................................................   60      1   Fm3
....................ugagguaguagguuAuauaguu....................................................................   20      1   Fm3
....................ugagguaguagguugAauaguu....................................................................   14      1   Fm3
....................ugagguaguagguuguauUguu....................................................................   15      1   Fm3
....................ugaCguaguagguuguauaguu....................................................................   9       1   Fm3
....................ugagguaUuagguuguauaguu....................................................................   11      1   Fm3
....................ugaggCaguagguuguauaguu....................................................................   24      1   Fm3
....................ugagguaguaggCuguauaguu....................................................................   35      1   Fm3
....................ugagguaguagguGguauaguu....................................................................   11      1   Fm3
....................ugagguaguaggAuguauaguu....................................................................   9       1   Fm3
....................ugagguagCagguuguauaguu....................................................................   25      1   Fm3
....................ugaggAaguagguuguauaguu....................................................................   22      1   Fm3
....................ugagguagCuguauaguu....................................................................   7       1   Fm3
....................ugagguaguagguuguauagGu....................................................................   2       1   Fm3
....................uCagguaguagguuguauaguu....................................................................   19      1   Fm3
....................ugagguaguaUguuguauaguu....................................................................   152     1   Fm3
....................ugUgguaguagguuguauaguu....................................................................   98      1   Fm3
....................ugagguCguagguuguauaguu....................................................................   22      1   Fm3
....................ugagUuaguagguuguauaguu....................................................................   5       1   Fm3
....................ugagguaguagguugGauaguu....................................................................   4       1   Fm3
....................ugaUguaguagguuguauaguu....................................................................   29      1   Fm3
....................ugagguaguagguuguauCguu....................................................................   8       1   Fm3
....................ugagguaguagguuguauGguu....................................................................   12      1   Fm3
....................ugagguaguagguuguUuaguu....................................................................   146     1   Fm3
....................ugagguaguagguuguauaUuu....................................................................   16      1   Fm3
....................uUagguaguagguuguauaguu....................................................................   40      1   Fm3
....................ugagguaguagguuguauagCu....................................................................   9       1   Fm3
....................Ggagguaguagguuguauaguu....................................................................   14      1   Fm3
....................ugagguaguagguuCuauaguu....................................................................   2       1   Fm3
....................ugagguaguagguuguaAaguu....................................................................   593     1   Fm3
....................ugaAguaguagguuguauaguu....................................................................   18      1   Fm3
....................ugagguaguagguuguauaguG....................................................................   23      1   Fm3
....................ugagguaguCgguuguauaguu....................................................................   15      1   Fm3
....................ugagguaguaAguuguauaguu....................................................................   230     1   Fm3
....................ugagguaguagguCguauaguu....................................................................   44      1   Fm3
....................ugagguaguagguuguauaguuC...................................................................   46      1   Fm3
....................ugagguaguagguuguauaguuU...................................................................   11990   1   Fm3
....................ugagguaguagguuguauaguAg...................................................................   5       1   Fm3
....................ugagguaguagguuguauaguug...................................................................   96      0   Fm3
....................ugagguaguagguuAuauaguug...................................................................   1       1   Fm3
....................ugagguaguagguuguauaguuA...................................................................   898     1   Fm3
....................ugagguaguagguuguauaguugu..................................................................   23      0   Fm3
....................ugagguaguagguuguauaguuAu..................................................................   32      1   Fm3
....................ugagguaguagguuguauaguuCu..................................................................   7       1   Fm3
....................ugagguaguagguuguauaguuUu..................................................................   74      1   Fm3
....................ugagguaguagguuguauaguuguA.................................................................   1       1   Fm3
....................ugagguaguagguuguauaguugugu................................................................   4       0   Fm3
....................ugagguaguagguuguauaguuguguac..............................................................   2       0   Fm3
....................ugagguaguagguuguauaguuguguacu.............................................................   3       0   Fm3
.....................gagguaguagguuguauU.......................................................................   3       1   Fm3
.....................gagguaguagguuguauag......................................................................   5       0   Fm3
.....................Uagguaguagguuguauag......................................................................   1       1   Fm3
.....................gagguUguagguuguauagu.....................................................................   1       1   Fm3
.....................gagguagAagguuguauagu.....................................................................   1       1   Fm3
```

```
                    Mature                                      Star
caggagugggccccucagucugagguaguagguuguauaguuguguacuacaucaucuuggggaacuguacaaccuucuagcuuucccagagggcgccucaguacaacau
.....................gagguaguagguuguauagu.....................................................................   21    0   Fm3
.....................Uagguaguagguuguauaguu....................................................................   2     1   Fm3
.....................gagguaguagguuguauaguu....................................................................   53    0   Fm3
.....................Nagguaguagguuguauaguu....................................................................   1     1   Fm3
.....................gagguaguagUuuguauaguu....................................................................   1     1   Fm3
.....................gagguaguagguuguCuaguu....................................................................   1     1   Fm3
.....................gagguaguagguuguauaguug...................................................................   1     0   Fm3
.....................gagguaguagguuguauaguuU...................................................................   6     1   Fm3
......................agguaguagguuguauaU......................................................................   1     1   Fm3
......................Ugguaguagguuguauaguu....................................................................   3     1   Fm3
......................agguaguagguuguauaguu....................................................................   3     0   Fm3
......................agguaguagguuguauaguuU...................................................................   1     1   Fm3
......................agguaguagguuguauaguuguUu................................................................   1     1   Fm3
.......................gguaguagguuguauaAuu....................................................................   1     1   Fm3
.......................gguaguagguuguauaguu....................................................................   11    0   Fm3
.........................uaguagguuguauaguuU...................................................................   1     1   Fm3
.........................uaguagguuguauaguuguguacu.............................................................   2     0   Fm3
.........................................uguguacuacaucaucuugggga..............................................   5     0   Fm3
................................................uacaucaucuuggggaacuguacaacc...................................   2     0   Fm3
.......................................................ucuuggggaacuguacaaccuucuag.............................   1     0   Fm3
.........................................................uuggggaacuguacaaccuucu...............................   1     0   Fm3
.................................................................cuguacaaccuucuagcu...........................   3     0   Fm3
.................................................................cuguacaaccuucuagcuu..........................   1     0   Fm3
.................................................................cuguacaaccuucuagcuuuc........................   2     0   Fm3
.................................................................cuguacaaccuucuagcuuucU.......................   1     1   Fm3
.................................................................Uuguacaaccuucuagcuuucc.......................   1     1   Fm3
.................................................................cuguacaaccGucuagcuuucc.......................   1     1   Fm3
.................................................................cuguacaaccAucuagcuuucc.......................   1     1   Fm3
.................................................................cuguacaaccuucuagcCuucc.......................   1     1   Fm3
.................................................................cuguacaaccuucuagcuuucc.......................   46    0   Fm3
.................................................................cuguacaaccuucuagcuuuccU......................   3     1   Fm3
.................................................................cuguacaaccuucuagcuuuccA......................   11    1   Fm3
..................................................................uguacaaccuucuagcuuuccU......................   1     1   Fm3

..................Ccugagguaguagguuguauaguu....................................................................   4     1   Mm1
...................cugagguaguagguuguauaguu....................................................................   14    0   Mm1
....................ugagguaguagguuguau........................................................................   137   0   Mm1
....................ugagguaguagguuguCu........................................................................   1     1   Mm1
....................Ngagguaguagguuguau........................................................................   1     1   Mm1
....................ugagguaguGgguuguau........................................................................   9     1   Mm1
....................ugCgguaguagguuguau........................................................................   1     1   Mm1
....................ugagguaguagguuguaA........................................................................   13    1   Mm1
....................ugGgguaguagguuguau........................................................................   1     1   Mm1
....................ugagguaguagguuguUu........................................................................   13    1   Mm1
....................ugagAuaguagguuguau........................................................................   1     1   Mm1
....................ugagguaguagguuguaG........................................................................   1     1   Mm1
....................ugagguaguagguuguaCa.......................................................................   1     1   Mm1
....................ugagguaguagguuguaGa.......................................................................   2     1   Mm1
....................ugagguaguagguuguauU.......................................................................   101   1   Mm1
....................ugagguaguagguuguaAa.......................................................................   7     1   Mm1
....................ugagguaguagguuguaua.......................................................................   99    0   Mm1
....................ugagguaguaUguuguauag......................................................................   1     1   Mm1
....................ugGgguaguagguuguauag......................................................................   1     1   Mm1
....................ugagguaguagguuguGuag......................................................................   1     1   Mm1
....................ugagguaguagguuguaGag......................................................................   5     1   Mm1
....................ugagguUguagguuguauag......................................................................   1     1   Mm1
....................ugagguaguagguuguauaU......................................................................   27    1   Mm1
....................ugagguaguaCguuguauag......................................................................   1     1   Mm1
....................ugagguaguagguuguaCag......................................................................   7     1   Mm1
....................ugagguaguagguuguCuag......................................................................   5     1   Mm1
....................ugagguaguagguugCauag......................................................................   1     1   Mm1
....................ugagguaguagguuguUuag......................................................................   2     1   Mm1
....................ugagguaguagguuUuauag......................................................................   1     1   Mm1
....................Ngagguaguagguuguauag......................................................................   2     1   Mm1
....................ugagguaguagguuguaAag......................................................................   8     1   Mm1
....................ugagguaguagguuguauag......................................................................   837   0   Mm1
....................ugagguaguaAguuguauag......................................................................   2     1   Mm1
....................ugagguaguagguuguauaUu.....................................................................   2     1   Mm1
....................ugagguaguUgguuguauagu.....................................................................   1     1   Mm1
....................ugaggNaguagguuguauagu.....................................................................   1     1   Mm1
```

Mature Star

```
caggagugggccccucagucugagguaguagguuguauaguuguguacuacaucaucuuggggaacuguacaaccuucuagcuuucccagagggcgccucaguacaacau
....................Cgagguaguagguuguauagu.....................................................................   1       1   Mm1
....................ugagguaguaCguuguauagu.....................................................................   7       1   Mm1
....................ugagguaguagguAguauagu.....................................................................   2       1   Mm1
....................ugCgguaguagguuguauagu.....................................................................   7       1   Mm1
....................ugagguaguagguuguauagA.....................................................................   30      1   Mm1
....................ugaggAaguagguuguauagu.....................................................................   2       1   Mm1
....................Agagguaguagguuguauagu.....................................................................   1       1   Mm1
....................ugagguaguagguuguaAagu.....................................................................   44      1   Mm1
....................ugagguaguagguugAauagu.....................................................................   1       1   Mm1
....................uAagguaguagguuguauagu.....................................................................   4       1   Mm1
....................ugagguaguagguuguCuagu.....................................................................   17      1   Mm1
....................ugagguaguagguuguauagG.....................................................................   1       1   Mm1
....................ugagguaguaUguuguauagu.....................................................................   7       1   Mm1
....................ugagguNguagguuguauagu.....................................................................   2       1   Mm1
....................ugGgguaguagguuguauagu.....................................................................   7       1   Mm1
....................ugagguaguaAguuguauagu.....................................................................   10      1   Mm1
....................ugagguGguagguuguauagu.....................................................................   1       1   Mm1
....................ugagguaguagguuguGuagu.....................................................................   7       1   Mm1
....................ugagguaguagUuuguauagu.....................................................................   1       1   Mm1
....................ugagguaguagguuguauGgu.....................................................................   2       1   Mm1
....................ugagguaguagguCguauagu.....................................................................   2       1   Mm1
....................ugagguaguagguuguaGagu.....................................................................   37      1   Mm1
....................uUagguaguagguuguauagu.....................................................................   1       1   Mm1
....................ugagguaguagguuguaCagu.....................................................................   38      1   Mm1
....................ugagguaguagguuguUuagu.....................................................................   7       1   Mm1
....................ugagguaguagAuuguauagu.....................................................................   6       1   Mm1
....................ugaUguaguagguuguauagu.....................................................................   1       1   Mm1
....................ugagguaguagguuguauagu.....................................................................   7077    0   Mm1
....................ugUgguaguagguuguauagu.....................................................................   9       1   Mm1
....................ugagguagCagguuguauagu.....................................................................   4       1   Mm1
....................ugaggCaguagguuguauagu.....................................................................   1       1   Mm1
....................ugagguUguagguuguauagu.....................................................................   2       1   Mm1
....................Ngagguaguagguuguauagu.....................................................................   2       1   Mm1
....................ugagguaguagguuguauUgu.....................................................................   1       1   Mm1
....................ugagguaUuagguuguauagu.....................................................................   11      1   Mm1
....................ugagguaguGgguuguauaguu....................................................................   5       1   Mm1
....................ugagguaguagguuguauagAu....................................................................   4       1   Mm1
....................uAagguaguagguuguauaguu....................................................................   6       1   Mm1
....................ugagAuaguagguuguauaguu....................................................................   3       1   Mm1
....................ugagguaguagUuuguauaguu....................................................................   3       1   Mm1
....................ugagguaguagguAguauaguu....................................................................   2       1   Mm1
....................ugagguUguagguuguauaguu....................................................................   9       1   Mm1
....................Cgagguaguagguuguauaguu....................................................................   11      1   Mm1
....................ugagguaguagguuUuauaguu....................................................................   2       1   Mm1
....................ugagguaguUgguuguauaguu....................................................................   2       1   Mm1
....................ugagguagGagguuguauaguu....................................................................   1       1   Mm1
....................ugagguaguagguuguauUguu....................................................................   2       1   Mm1
....................ugagguGguagguuguauaguu....................................................................   9       1   Mm1
....................Ngagguaguagguuguauaguu....................................................................   14      1   Mm1
....................uUagguaguagguuguauaguu....................................................................   12      1   Mm1
....................ugagguaguagguuguaCaguu....................................................................   167     1   Mm1
....................ugaAguaguagguuguauaguu....................................................................   1       1   Mm1
....................ugagguaguCgguuguauaguu....................................................................   1       1   Mm1
....................ugagguaguaAguuguauaguu....................................................................   51      1   Mm1
....................ugagguaguaggAuguauaguu....................................................................   2       1   Mm1
....................ugagguaguaCguuguauaguu....................................................................   23      1   Mm1
....................ugagguaguagguuguUuaguu....................................................................   34      1   Mm1
....................ugagguaUuagguuguauaguu....................................................................   5       1   Mm1
....................ugagguCguagguuguauaguu....................................................................   1       1   Mm1
....................ugaggGaguagguuguauaguu....................................................................   2       1   Mm1
....................ugagguaguagguuguauaAuu....................................................................   1       1   Mm1
....................ugagguaguagguuguauaguu....................................................................   27180   0   Mm1
....................ugaggAaguagguuguauaguu....................................................................   3       1   Mm1
....................ugagguaguagguuguauaUuu....................................................................   1       1   Mm1
....................ugagguaguagguuAuauaguu....................................................................   3       1   Mm1
....................ugagguaguagguuguauaguC....................................................................   1       1   Mm1
....................ugGgguaguagguuguauaguu....................................................................   38      1   Mm1
....................ugagguaguagguuguGuaguu....................................................................   25      1   Mm1
....................ugagguaguaUguuguauaguu....................................................................   27      1   Mm1
....................ugagUuaguagguuguauaguu....................................................................   1       1   Mm1
```

Mature **Star**

```
caggagugggccccucagucugagguaguagguuguauaguuguguacuacaucaucuuggggaacuguacaaccuucuagcuuucccagagggcgccucaguacaacau
....................ugagguaguagAuuguauaguu....................................................................    1      1    Mm1
....................ugagguaguagguuguaAaguu....................................................................    131    1    Mm1
....................ugagguagCagguuguauaguu....................................................................    1      1    Mm1
....................ugagguaguagguuguauagCu....................................................................    1      1    Mm1
....................ugaggCaguagguuguauaguu....................................................................    5      1    Mm1
....................ugagguaguagguuguCuaguu....................................................................    64     1    Mm1
....................ugaUguaguagguuguauaguu....................................................................    2      1    Mm1
....................ugagguaguagguuguauaguA....................................................................    241    1    Mm1
....................ugagCuaguagguuguauaguu....................................................................    2      1    Mm1
....................ugCgguaguagguuguauaguu....................................................................    23     1    Mm1
....................ugagguaguagguugCauaguu....................................................................    4      1    Mm1
....................uNagguaguagguuguauaguu....................................................................    1      1    Mm1
....................ugagguaguagguugAauaguu....................................................................    1      1    Mm1
....................ugagguaguagguuguauaguG....................................................................    4      1    Mm1
....................ugagguaguagguGguauaguu....................................................................    4      1    Mm1
....................ugagguaguagguuguauCguu....................................................................    3      1    Mm1
....................Ggagguaguagguuguauaguu....................................................................    1      1    Mm1
....................ugagguagAagguuguauaguu....................................................................    1      1    Mm1
....................ugagguaguaggGuguauaguu....................................................................    1      1    Mm1
....................ugaCguaguagguuguauaguu....................................................................    1      1    Mm1
....................ugagguaguaggCuguauaguu....................................................................    3      1    Mm1
....................ugaggNaguagguuguauaguu....................................................................    1      1    Mm1
....................ugagguaguagguCguauaguu....................................................................    7      1    Mm1
....................Agagguaguagguuguauaguu....................................................................    9      1    Mm1
....................ugagguaguagguuguaGaguu....................................................................    143    1    Mm1
....................ugUgguaguagguuguauaguu....................................................................    19     1    Mm1
....................ugagguaguagguuguauGguu....................................................................    2      1    Mm1
....................ugagguaguagguuguauaguuA...................................................................    219    1    Mm1
....................ugagguaguagguuguUuaguug...................................................................    1      1    Mm1
....................ugagguaguagguuguauaguuU...................................................................    2133   1    Mm1
....................ugagguaguagguuguauaguug...................................................................    11     0    Mm1
....................ugagguaguUgguuguauaguug...................................................................    1      1    Mm1
....................ugagguaguagguuguauaguuUu..................................................................    59     1    Mm1
....................ugagguaguagguuguauaguuAu..................................................................    8      1    Mm1
....................ugagguaguagguuguauaguuguguaU..............................................................    1      1    Mm1
.....................gagguaguCgguuguauagu.....................................................................    1      1    Mm1
.....................gagguaguagguuguauagu.....................................................................    19     0    Mm1
.....................gagguaguagguuguauaguu....................................................................    10     0    Mm1
.....................gagguaguagguuguauaguuU...................................................................    1      1    Mm1
........................................................cuuggggaacuguacaaccuucuagcuuucc.......................    3      0    Mm1
..........................................................uggggaacuguacaaccuucuagcuuucc.......................    5      0    Mm1
............................................................gggaacuguacaaccuucuagc............................    3      0    Mm1
.................................................................cuguacaaccuucuagcu...........................    11     0    Mm1
.................................................................cuguacaaccuucuagcuu..........................    7      0    Mm1
.................................................................cuguacaaccuucuagAuuuc........................    1      1    Mm1
.................................................................cuguacaaccuucuagcuuuc........................    43     0    Mm1
.................................................................cuguacaaccuucuagcAuuc........................    1      1    Mm1
.................................................................cuguacaaccuucuagcCuuc........................    1      1    Mm1
.................................................................cuguaAaaccuucuagcuuucc.......................    1      1    Mm1
.................................................................cuguacaaccuucuagcCuucc.......................    6      1    Mm1
.................................................................cuguacaaccGucuagcuuucc.......................    1      1    Mm1
.................................................................cuguacaaccuucuagUuuucc.......................    2      1    Mm1
.................................................................cuguacaaccuucuagcuuucc.......................    1099   0    Mm1
.................................................................cuguacaGccuucuagcuuucc.......................    1      1    Mm1
.................................................................cuguacaaccuucuagAuuucc.......................    8      1    Mm1
.................................................................cuguacUaccuucuagcuuucc.......................    1      1    Mm1
.................................................................Uuguacaaccuucuagcuuucc.......................    7      1    Mm1
.................................................................cuguacaaccCucuagcuuucc.......................    2      1    Mm1
.................................................................Nuguacaaccuucuagcuuucc.......................    2      1    Mm1
.................................................................cuguacaaccuucuagcGuucc.......................    7      1    Mm1
.................................................................cuguacaaccuucuagcAuucc.......................    5      1    Mm1
.................................................................cuguacaaccAucuagcuuucc.......................    1      1    Mm1
.................................................................cuguaUaaccuucuagcuuucc.......................    1      1    Mm1
.................................................................cuguacaaccuucuagcuuuccA......................    32     1    Mm1
.................................................................cuguacaaccuucuagcuuuccU......................    45     1    Mm1

..................ucugagguaguagguuguauagu.....................................................................    7      0    Mm2
...................cugagguaguagguuguauagu.....................................................................    1      0    Mm2
...................cugagguaguagguuguauaguu....................................................................    3      0    Mm2
...................cugagguaguaggGuguauaguu....................................................................    1      1    Mm2
```

```
                    Mature                                            Star
caggagugggccccucagucugagguaguagguuguauaguuguguacuacaucaucuuggggaacuguacaaccuucuagcuuucccagagggcgccucaguacaacau
....................ugagguaguagguuguau........................................................................    78     0    Mm2
....................ugagguaguagguuguUu........................................................................    5      1    Mm2
....................ugagguaguagguuguaA........................................................................    6      1    Mm2
....................ugagguaguagguuguaG........................................................................    1      1    Mm2
....................ugagguaguagguuguaua.......................................................................    93     0    Mm2
....................ugagguaguGgguuguaua.......................................................................    1      1    Mm2
....................ugagguaguagguuguUua.......................................................................    1      1    Mm2
....................ugagguaguagguuguauU.......................................................................    81     1    Mm2
....................ugagguaguaggAuguaua.......................................................................    1      1    Mm2
....................ugagguaguagguuguaAa.......................................................................    15     1    Mm2
....................ugagguaguagguuguaCa.......................................................................    1      1    Mm2
....................ugagguaguagguuguauaU......................................................................    10     1    Mm2
....................ugagguaguagguuguaAag......................................................................    2      1    Mm2
....................ugagguaguagguCguauag......................................................................    1      1    Mm2
....................ugCgguaguagguuguauag......................................................................    1      1    Mm2
....................Ggagguaguagguuguauag......................................................................    2      1    Mm2
....................ugagguaguagCuuguauag......................................................................    1      1    Mm2
....................ugagguaguagguuguauag......................................................................    695    0    Mm2
....................ugagguaguagguuguaGag......................................................................    2      1    Mm2
....................Agagguaguagguuguauag......................................................................    1      1    Mm2
....................ugagguaguagguuguCuag......................................................................    2      1    Mm2
....................ugaggGaguagguuguauag......................................................................    1      1    Mm2
....................ugagguUguagguuguauag......................................................................    1      1    Mm2
....................ugagguaguagguuguauaA......................................................................    12     1    Mm2
....................ugagguaguagguuguaCag......................................................................    5      1    Mm2
....................ugagguaguaAguuguauag......................................................................    6      1    Mm2
....................ugagguaguagguuguGuag......................................................................    1      1    Mm2
....................ugagCuaguagguuguauag......................................................................    1      1    Mm2
....................ugagguaguagguuguUuag......................................................................    3      1    Mm2
....................ugagguagGagguuguauagu.....................................................................    2      1    Mm2
....................ugagguaguagguuguauagC.....................................................................    2      1    Mm2
....................uUagguaguagguuguauagu.....................................................................    4      1    Mm2
....................ugagguaguagguuAuauagu.....................................................................    1      1    Mm2
....................ugagguaguagguuguauagA.....................................................................    24     1    Mm2
....................ugagguaguagguuguauaCu.....................................................................    1      1    Mm2
....................Agagguaguagguuguauagu.....................................................................    1      1    Mm2
....................ugUgguaguagguuguauagu.....................................................................    3      1    Mm2
....................ugagguaguagguuUuauagu.....................................................................    2      1    Mm2
....................ugGgguaguagguuguauagu.....................................................................    10     1    Mm2
....................ugagguaguagguuguauagu.....................................................................    7052   0    Mm2
....................ugagguaguaUguuguauagu.....................................................................    8      1    Mm2
....................ugagguaguagguuguGuagu.....................................................................    11     1    Mm2
....................ugagguaguGgguuguauagu.....................................................................    1      1    Mm2
....................ugagguaguagguCguauagu.....................................................................    1      1    Mm2
....................ugagguaguagguuguauUgu.....................................................................    1      1    Mm2
....................ugagguaguagguugCauagu.....................................................................    2      1    Mm2
....................ugagguaguagguuguaGagu.....................................................................    44     1    Mm2
....................ugagguaguagguuguCuagu.....................................................................    18     1    Mm2
....................ugagguaguagguuguUuagu.....................................................................    11     1    Mm2
....................ugagguUguagguuguauagu.....................................................................    1      1    Mm2
....................ugagguaguUgguuguauagu.....................................................................    1      1    Mm2
....................Ngagguaguagguuguauagu.....................................................................    3      1    Mm2
....................ugagguaguagguuguaAagu.....................................................................    21     1    Mm2
....................ugagguaguaCguuguauagu.....................................................................    5      1    Mm2
....................ugagguaAuagguuguauagu.....................................................................    1      1    Mm2
....................ugagguaguaAguuguauagu.....................................................................    7      1    Mm2
....................ugagguaguagguuguauaUu.....................................................................    1      1    Mm2
....................ugagguaguagguuguauagG.....................................................................    5      1    Mm2
....................Cgagguaguagguuguauagu.....................................................................    1      1    Mm2
....................ugagCuaguagguuguauagu.....................................................................    1      1    Mm2
....................Ggagguaguagguuguauagu.....................................................................    2      1    Mm2
....................ugagguaguagguuguauCgu.....................................................................    1      1    Mm2
....................ugagguaguagguuguaCagu.....................................................................    39     1    Mm2
....................uAagguaguagguuguauagu.....................................................................    1      1    Mm2
....................ugagguaUuagguuguauagu.....................................................................    1      1    Mm2
....................ugagguaguaggCuguauagu.....................................................................    1      1    Mm2
....................ugCgguaguagguuguauagu.....................................................................    4      1    Mm2
....................ugagguaguagguugAauagu.....................................................................    2      1    Mm2
....................ugagguaguagguAguauagu.....................................................................    2      1    Mm2
....................ugagguaguagguuguCuaguu....................................................................    44     1    Mm2
```

Mature **Star**

caggagugggccccucagucugagguaguagguuguauaguuguguacuacaucaucuuggggaacuguacaaccuucuagcuuucccagagggcgccucaguacaacau			
....................ugagguaguagguuguaGaguu..	95	1	Mm2
....................Agagguaguagguuguauaguu..	7	1	Mm2
....................ugagguaguagguuguaCaguu..	119	1	Mm2
....................ugagguagGagguuguauaguu..	1	1	Mm2
....................ugaAguaguagguuguauaguu..	4	1	Mm2
....................ugagguaguagguuguauGguu..	3	1	Mm2
....................ugagguaguagCuuguauaguu..	2	1	Mm2
....................Cgagguaguagguuguauaguu..	7	1	Mm2
....................ugaUguaguagguuguauaguu..	2	1	Mm2
....................ugagguaguaggAuguauaguu..	2	1	Mm2
....................ugagguaguagguuguaAaguu..	89	1	Mm2
....................ugUgguaguagguuguauaguu..	9	1	Mm2
....................ugagguaguagguuguUuaguu..	18	1	Mm2
....................ugagguUguagguuguauaguu..	7	1	Mm2
....................ugagguGguagguuguauaguu..	5	1	Mm2
....................ugaggNaguagguuguauaguu..	1	1	Mm2
....................ugagguaguagguuguauaguu..	19324	0	Mm2
....................ugagguaguagguuguGuaguu..	28	1	Mm2
....................ugagguaUuagguuguauaguu..	1	1	Mm2
....................ugagguaguUgguuguauaguu..	6	1	Mm2
....................ugagguaguagguuguauaUuu..	2	1	Mm2
....................ugagguaguagguAguauaguu..	3	1	Mm2
....................ugCgguaguagguuguauaguu..	13	1	Mm2
....................ugagguaguGgguuguauaguu..	11	1	Mm2
....................ugagguaguaggCuguauaguu..	2	1	Mm2
....................ugagguaguagguUuauaguu...	1	1	Mm2
....................ugagguaguaUguuguauaguu..	22	1	Mm2
....................uCagguaguagguuguauaguu..	3	1	Mm2
....................ugagguaguCgguuguauaguu..	3	1	Mm2
....................ugagguaguagguuguauagGu..	3	1	Mm2
....................ugagguaguagUuuguauaguu..	4	1	Mm2
....................ugagguaguagguuguauUguu..	5	1	Mm2
....................ugagguaguagguuguauaguC..	2	1	Mm2
....................Ggagguaguagguuguauaguu..	4	1	Mm2
....................ugagguaguaAguuguauaguu..	28	1	Mm2
....................ugagguaguagguuguauaCuu..	3	1	Mm2
....................ugagguaguaggGuguauaguu..	2	1	Mm2
....................ugagguaguagguuguauaAuu..	3	1	Mm2
....................ugagguaguagguuguauaguA..	147	1	Mm2
....................uAagguaguagguuguauaguu..	6	1	Mm2
....................ugagguaguagguCguauaguu..	2	1	Mm2
....................ugagguagAagguuguauaguu..	1	1	Mm2
....................ugagguagCagguuguauaguu..	1	1	Mm2
....................ugagguaguagguuguauCguu..	1	1	Mm2
....................ugagguaguaCguuguauaguu..	23	1	Mm2
....................ugagguCguagguuguauaguu..	2	1	Mm2
....................ugaggGaguagguuguauaguu..	2	1	Mm2
....................ugagguNguagguuguauaguu..	3	1	Mm2
....................ugagguaguagguuguauaguG..	16	1	Mm2
....................ugagguaguagguuguauagAu..	4	1	Mm2
....................Ngagguaguagguuguauaguu..	7	1	Mm2
....................ugaggCaguagguuguauaguu..	2	1	Mm2
....................ugGgguaguagguuguauaguu..	25	1	Mm2
....................ugagguaAuagguuguauaguu..	1	1	Mm2
....................ugagguaguagguuguauaguug...	19	0	Mm2
....................ugagguaguagguuguauaguAg...	7	1	Mm2
....................ugagguaguagguuguauaguuC...	14	1	Mm2
....................ugagguaguagguuguauaguuA...	297	1	Mm2
....................ugagguaguagguuguauaguuU...	1843	1	Mm2
....................ugagguaguagguuguauaguuUu..	88	1	Mm2
....................ugagguaguagguuguauaguugA..	1	1	Mm2
....................ugagguaguagguuguauaguugu..	4	0	Mm2
....................ugagguaguagguuguauaguuAu..	19	1	Mm2
....................ugagguaguagguuguauaguuCu..	4	1	Mm2
....................ugagguaguagguuguauaguugug...	1	0	Mm2
....................ugagguaguagguuguauaguuguguac..	1	0	Mm2
....................ugagguaguagguuguauaguuguguacu...	2	0	Mm2
.....................gagguaguagguuguauag..	1	0	Mm2
.....................gagguaguagguuguauaguu..	6	0	Mm2
.....................gagguaguagguuguauaguugA..	1	1	Mm2

Mature **Star**

caggagugggccccucagucugagguaguagguuguauaguuguguacuacaucaucuuggggaacuguacaaccuucuagcuuucccagagggcgccucaguacaacau			
....uguguacuacaucaucuuggggaac..	1	0	Mm2
....guguacuacaucaucuugggg...	1	0	Mm2
....guguacuacaucaucuuggggga..	1	0	Mm2
....guguacuacaucaucuuggggaa...	1	0	Mm2
....acaucaucuuggggaacuguacaaccuucuagcu...........................	1	0	Mm2
....acaucaucuuggggaacuguacaaccuucuagcuuucc.......................	1	0	Mm2
....aucuuggggaacuguacaaccuucuagcuuucc.......................	2	0	Mm2
....aucuuggggaacuguacaaccuucuagcuuuccU......................	1	1	Mm2
....ucuuggggaacuguacaaccu..................................	1	0	Mm2
....ucuuggggaacuguacaaccuucuagcu...........................	1	0	Mm2
....cuuggggaacuguacaaccuucuagcuuucc.......................	5	0	Mm2
....uuggggaacuguacaaccuucu...............................	6	0	Mm2
....uuggggaacuguacaaccuucuagcuuuc........................	2	0	Mm2
....uuggggaacuguacaaccuucuagcuuucc.......................	10	0	Mm2
....uuggggaacuguacaaccuucuagcuuuccU......................	1	1	Mm2
....uggggaacuguacaaccuucuagcuuuc........................	1	0	Mm2
....uggggaacuguacaaccuucuagcuuucc.......................	8	0	Mm2
....ggggaacuguacaaccuucu...............................	1	0	Mm2
....ggggaacuguacaaccuucuagcuuucc.......................	3	0	Mm2
....ggggaacuguUcaaccuucuagcuuucc.......................	1	1	Mm2
....ggggaacuguacaaccuucuagcuuuccU......................	1	1	Mm2
....gaacuguacaaccuucuagcuuucU.......................	1	1	Mm2
....aacuguacaaccuucuagcuuucc.......................	1	0	Mm2
....acuguacaaccuucuagcuuucc.......................	1	0	Mm2
....cuguacaaccuucuagcu...........................	1	0	Mm2
....cuguacaaccuucuagcuuu.........................	1	0	Mm2
....cuguacaaccuucuagcuuuc........................	35	0	Mm2
....cuguacaaccuucuagcuuuUc.......................	1	1	Mm2
....cuguaUaaccuucuagcuuucc.......................	1	1	Mm2
....cugCacaaccuucuagcuuucc.......................	1	1	Mm2
....cuguacaaccuucuagcAuucc.......................	3	1	Mm2
....cuguacaaccuucuagcGuucc.......................	2	1	Mm2
....cuguacaaccuucuagAuuucc.......................	2	1	Mm2
....cuguacaaccCucuagcuuucc.......................	1	1	Mm2
....cuguacaaccuucuagcuuucU.......................	17	1	Mm2
....cuguacaaccuucuagcCuucc.......................	1	1	Mm2
....cuguacaaccuucuagUuuucc.......................	1	1	Mm2
....cuguacaaccGucuagcuuucc.......................	1	1	Mm2
....cuguacaaccuucuagcuuucc.......................	757	0	Mm2
....cuguacaGccuucuagcuuucc.......................	1	1	Mm2
....cuguacaaccuucuagcuCucc.......................	1	1	Mm2
....cuguacaaccAucuagcuuucc.......................	1	1	Mm2
....cAguacaaccuucuagcuuucc.......................	1	1	Mm2
....Auguacaaccuucuagcuuucc.......................	1	1	Mm2
....cuguacaaccuucuagcuuuccU......................	27	1	Mm2
....cuguacaaccuucuagcuuuccG......................	1	1	Mm2
....cuguacaaccuucuagcuuuccA......................	70	1	Mm2
....cuguacaaccuucuagcuuuccc......................	1	0	Mm2
....uguacaaccuucuagcuuucc.......................	14	0	Mm2
....uguacaaccuucuagcuuuccU......................	5	1	Mm2
....uguacaaccuucuagcuuuccA......................	5	1	Mm2
..................ucugagguaguagguuguauag..	21	0	Fm2
..................ucugagguaguagguuguauagG...	1	1	Fm2
..................ucugagguGguagguuguauagu...	1	1	Fm2
..................ucugagguaguagguuguauagu...	87	0	Fm2
..................ucugagguaguagguugCauagu...	1	1	Fm2
..................ucugagguaguagguuguauaguu..	2	0	Fm2
...................cugagguaguagguuguauagu...	4	0	Fm2
...................cugagguaguagguuguauaUu...	1	1	Fm2
...................cugaggAaguagguuguauaguu..	2	1	Fm2
...................cugagguaguagguuguauaguu..	10	0	Fm2
....................ugagguaguagguuguaA..	3	1	Fm2
....................ugagguaguagguuguCu..	1	1	Fm2
....................ugagguaguagguuguUu..	9	1	Fm2
....................ugagguaguagguuguGu..	1	1	Fm2
....................ugagguaguagguuguau..	94	0	Fm2
....................ugagguaguagguuguaCa...	2	1	Fm2
....................ugagguaguagguuguauU...	130	1	Fm2
....................ugagguaguagguuguaua...	233	0	Fm2

Mature Star

```
caggagugggccccucagucugagguaguagguuguauaguuguguacuacaucaucuuggggaacuguacaaccuucuagcuuucccagagggcgccucaguacaacau
....................ugagguaguagguuguaGa.......................................................................   1      1   Fm2
....................ugagguaguagguuguUua.......................................................................   1      1   Fm2
....................ugagguaguagguuguaAa.......................................................................   13     1   Fm2
....................ugagguaguaCguuguaua.......................................................................   2      1   Fm2
....................ugagguaguaCguuguauag......................................................................   1      1   Fm2
....................ugagguaguagguuguauaU......................................................................   10     1   Fm2
....................uUagguaguagguuguauag......................................................................   3      1   Fm2
....................ugagguaguagguugGauag......................................................................   1      1   Fm2
....................ugagguaguagguuguaAag......................................................................   3      1   Fm2
....................ugagguaguagguuguaGag......................................................................   5      1   Fm2
....................uAagguaguagguuguauag......................................................................   1      1   Fm2
....................ugagguaguagguuguCuag......................................................................   4      1   Fm2
....................ugagguaguaUguuguauag......................................................................   1      1   Fm2
....................ugagguaguaAguuguauag......................................................................   3      1   Fm2
....................ugagguaguagguuguaCag......................................................................   6      1   Fm2
....................ugagguaguagguuguauag......................................................................   956    0   Fm2
....................ugagguaguagguuguauaA......................................................................   4      1   Fm2
....................ugagguaguUgguuguauag......................................................................   1      1   Fm2
....................Agagguaguagguuguauag......................................................................   2      1   Fm2
....................ugagguaguagguGguauag......................................................................   1      1   Fm2
....................ugagguaguagguuguGuag......................................................................   1      1   Fm2
....................Ngagguaguagguuguauag......................................................................   1      1   Fm2
....................ugagguagGagguuguauag......................................................................   1      1   Fm2
....................ugagguaguagguugAauagu.....................................................................   5      1   Fm2
....................ugagguagGagguuguauagu.....................................................................   5      1   Fm2
....................uAagguaguagguuguauagu.....................................................................   7      1   Fm2
....................ugagguaguagguAguauagu.....................................................................   5      1   Fm2
....................ugagguaguaAguuguauagu.....................................................................   31     1   Fm2
....................ugaCguaguagguuguauagu.....................................................................   1      1   Fm2
....................ugaggAaguagguuguauagu.....................................................................   1      1   Fm2
....................Ggagguaguagguuguauagu.....................................................................   2      1   Fm2
....................ugagguaguGgguuguauagu.....................................................................   5      1   Fm2
....................Ngagguaguagguuguauagu.....................................................................   9      1   Fm2
....................ugagguaguaUguuguauagu.....................................................................   12     1   Fm2
....................ugagguagAagguuguauagu.....................................................................   2      1   Fm2
....................ugagguaguagguuguaAagu.....................................................................   69     1   Fm2
....................ugagguaguUgguuguauagu.....................................................................   6      1   Fm2
....................ugagguUguagguuguauagu.....................................................................   4      1   Fm2
....................ugagguaguagguuguauaAu.....................................................................   2      1   Fm2
....................ugagguaguagguuAuauagu.....................................................................   4      1   Fm2
....................Agagguaguagguuguauagu.....................................................................   1      1   Fm2
....................ugagguNguagguuguauagu.....................................................................   1      1   Fm2
....................ugagguaguagguuguauagA.....................................................................   51     1   Fm2
....................ugagguaguagguuguCuagu.....................................................................   43     1   Fm2
....................ugagguaguaggCuguauagu.....................................................................   3      1   Fm2
....................ugagguaguaCguuguauagu.....................................................................   15     1   Fm2
....................ugUgguaguagguuguauagu.....................................................................   9      1   Fm2
....................ugagguaguagguuguauaUu.....................................................................   1      1   Fm2
....................uUagguaguagguuguauagu.....................................................................   2      1   Fm2
....................ugagUuaguagguuguauagu.....................................................................   1      1   Fm2
....................ugagguaguagguuguauagu.....................................................................   17065  0   Fm2
....................ugagguaguagCuuguauagu.....................................................................   2      1   Fm2
....................ugagguaguagguuguauagC.....................................................................   6      1   Fm2
....................ugagguGguagguuguauagu.....................................................................   4      1   Fm2
....................ugagguaguagguuguGuagu.....................................................................   15     1   Fm2
....................uCagguaguagguuguauagu.....................................................................   2      1   Fm2
....................ugagguaguagguuguaGagu.....................................................................   86     1   Fm2
....................ugagguagCagguuguauagu.....................................................................   1      1   Fm2
....................ugagCuaguagguuguauagu.....................................................................   1      1   Fm2
....................Cgagguaguagguuguauagu.....................................................................   3      1   Fm2
....................ugagguaguagUuuguauagu.....................................................................   3      1   Fm2
....................ugagguaguagguuguauUgu.....................................................................   2      1   Fm2
....................ugagAuaguagguuguauagu.....................................................................   2      1   Fm2
....................ugaUguaguagguuguauagu.....................................................................   1      1   Fm2
....................ugagguaguagguuCuauagu.....................................................................   1      1   Fm2
....................ugagguaguagguCguauagu.....................................................................   2      1   Fm2
....................ugCgguaguagguuguauagu.....................................................................   4      1   Fm2
....................ugagguaguagguugCauagu.....................................................................   3      1   Fm2
....................ugagguaguaggAuguauagu.....................................................................   2      1   Fm2
....................ugagguaguagguuguauagG.....................................................................   25     1   Fm2
```

```
                    Mature                                       Star
caggagugggccccucagucugagguaguagguuguauaguuguguacuacaucaucuuggggaacuguacaaccuucuagcuuucccagagggcgccucaguacaacau
....................ugaAguaguagguuguauagu.....................................................................    5      1    Fm2
....................ugaggGaguagguuguauagu.....................................................................    2      1    Fm2
....................ugagguaguagguuUuauagu.....................................................................    1      1    Fm2
....................ugagguaguagguuguaCagu.....................................................................    84     1    Fm2
....................ugGgguaguagguuguauagu.....................................................................    14     1    Fm2
....................ugagguaUuagguuguauagu.....................................................................    2      1    Fm2
....................ugagguaguagguuguUuagu.....................................................................    28     1    Fm2
....................ugagguUguagguuguauaguu....................................................................    20     1    Fm2
....................ugagguaguagguuguaAaguu....................................................................    257    1    Fm2
....................ugagguaguagguuCuauaguu....................................................................    2      1    Fm2
....................ugagguaguagguuAuauaguu....................................................................    13     1    Fm2
....................ugagguagCagguuguauaguu....................................................................    8      1    Fm2
....................ugagguaguagguuguauaAuu....................................................................    1      1    Fm2
....................ugagguaguagguuguauaUuu....................................................................    8      1    Fm2
....................ugagguaguaggGuguauaguu....................................................................    2      1    Fm2
....................ugagguaguagguugCauaguu....................................................................    4      1    Fm2
....................ugCgguaguagguuguauaguu....................................................................    32     1    Fm2
....................ugagguaguUgguuguauaguu....................................................................    7      1    Fm2
....................ugaAguaguagguuguauaguu....................................................................    5      1    Fm2
....................ugagguaguagguugAauaguu....................................................................    3      1    Fm2
....................ugagguaguagguuguauaguu....................................................................    60568  0    Fm2
....................Ggagguaguagguuguauaguu....................................................................    6      1    Fm2
....................ugUgguaguagguuguauaguu....................................................................    32     1    Fm2
....................ugagguaguagguuguauGguu....................................................................    8      1    Fm2
....................ugagguaguagguuguauagAu....................................................................    10     1    Fm2
....................ugaUguaguagguuguauaguu....................................................................    8      1    Fm2
....................ugagguaguGgguuguauaguu....................................................................    26     1    Fm2
....................uAagguaguagguuguauaguu....................................................................    21     1    Fm2
....................ugagguaguagguuguUuaguu....................................................................    71     1    Fm2
....................ugagguaguaAguuguauaguu....................................................................    93     1    Fm2
....................ugagguaguagguAguauaguu....................................................................    18     1    Fm2
....................ugagguaguagguuguCuaguu....................................................................    140    1    Fm2
....................ugagguaguagguGguauaguu....................................................................    3      1    Fm2
....................ugagguaguagguuguauaCuu....................................................................    1      1    Fm2
....................uUagguaguagguuguauaguu....................................................................    10     1    Fm2
....................ugagguaguagguuguaGaguu....................................................................    331    1    Fm2
....................ugagguaguagguCguauaguu....................................................................    23     1    Fm2
....................ugagguaguagguuguauCguu....................................................................    3      1    Fm2
....................ugagguagAagguuguauaguu....................................................................    17     1    Fm2
....................ugagguNguagguuguauaguu....................................................................    7      1    Fm2
....................ugagguaguagguuguauagGu....................................................................    6      1    Fm2
....................Cgagguaguagguuguauaguu....................................................................    15     1    Fm2
....................ugagUuaguagguuguauaguu....................................................................    5      1    Fm2
....................ugagguaguCgguuguauaguu....................................................................    2      1    Fm2
....................ugagguaguagCuuguauaguu....................................................................    1      1    Fm2
....................ugaggNaguagguuguauaguu....................................................................    8      1    Fm2
....................ugaggAaguagguuguauaguu....................................................................    12     1    Fm2
....................ugagguaguagguugGauaguu....................................................................    3      1    Fm2
....................ugagAuaguagguuguauaguu....................................................................    8      1    Fm2
....................ugagguCguagguuguauaguu....................................................................    3      1    Fm2
....................ugaCguaguagguuguauaguu....................................................................    2      1    Fm2
....................ugagguaguagAuuguauaguu....................................................................    3      1    Fm2
....................ugagguaguagguuguauaguG....................................................................    70     1    Fm2
....................ugagguaguagUuuguauaguu....................................................................    9      1    Fm2
....................ugagguaguaUguuguauaguu....................................................................    54     1    Fm2
....................uCagguaguagguuguauaguu....................................................................    5      1    Fm2
....................ugagguagGagguuguauaguu....................................................................    5      1    Fm2
....................ugagguaguagguuguauUguu....................................................................    13     1    Fm2
....................Ngagguaguagguuguauaguu....................................................................    28     1    Fm2
....................ugagguaguagguuguauagCu....................................................................    6      1    Fm2
....................ugaggGaguagguuguauaguu....................................................................    4      1    Fm2
....................ugagCuaguagguuguauaguu....................................................................    4      1    Fm2
....................Agagguaguagguuguauaguu....................................................................    15     1    Fm2
....................ugagguaUuagguuguauaguu....................................................................    5      1    Fm2
....................ugagguaguagguuguaCaguu....................................................................    328    1    Fm2
....................ugagguaguaggCuguauaguu....................................................................    12     1    Fm2
....................ugagguaguaCguuguauaguu....................................................................    54     1    Fm2
....................ugagguaguagguuguauaguA....................................................................    628    1    Fm2
....................ugagguaguagguuUuauaguu....................................................................    5      1    Fm2
....................ugaggCaguagguuguauaguu....................................................................    13     1    Fm2
```

Mature Star

caggagugggccccucagucugagguaguagguuguauaguuguguacuacaucaucuuggggaacuguacaaccuucuagcuuucccagagggcgccucaguacaacau			
....................ugagguGguagguuguauaguu..	11	1	Fm2
....................ugGgguaguagguuguauaguu..	75	1	Fm2
....................ugagguaguagguuguauaguC..	6	1	Fm2
....................ugagguaguagguuguGuaguu..	74	1	Fm2
....................ugagguaguaggAuguauaguu..	1	1	Fm2
....................ugagguaAuagguuguauaguu..	4	1	Fm2
....................ugagguUguagguuguauaguug...	1	1	Fm2
....................ugagguaguagguuguauaguuU...	4942	1	Fm2
....................ugagguaguagguuguauaguuC...	119	1	Fm2
....................ugagguaguagguuguaCaguug...	1	1	Fm2
....................ugagguaguagguuguauaguGg...	2	1	Fm2
....................ugagguaguagguuguauaguAg...	13	1	Fm2
....................ugagguaguagguuguauaguug...	68	0	Fm2
....................ugagguaguagguuguauaguuA...	609	1	Fm2
....................ugagguaguagguuguaAaguug...	2	1	Fm2
....................ugagguaguagguuguauaguuCu..	27	1	Fm2
....................ugagguaguagguuguauaguugu..	6	0	Fm2
....................ugagguaguagguuguauaguuAu..	24	1	Fm2
....................ugagguaguagguuguauaguuUu..	401	1	Fm2
.....................gagguaguagguuguauagu...	2	0	Fm2
.....................Uagguaguagguuguauaguu..	3	1	Fm2
.....................gagguaguagguuguauCguu..	1	1	Fm2
.....................gagguaguagguuguaCaguu..	1	1	Fm2
.....................gagguaguagguuguauaguu..	17	0	Fm2
.....................gagguaguagguuguauaguug...	9	0	Fm2
....ucuuggggaacuguacaaccuucuagcuuucc.......................	1	0	Fm2
....cuuggggaacuguacaacc...................................	5	0	Fm2
....uuggggaacuguacaaccuucu...............................	6	0	Fm2
....uuggggaacuguacaaccuuAuagcuuucc.......................	1	1	Fm2
....uuggggaacuguacaacUuucuagcuuucc.......................	1	1	Fm2
....uuggggaacuguacaaccuucuagcuuucc.......................	56	0	Fm2
....uuggggaacuguacaaUcuucuagcuuucc.......................	1	1	Fm2
....uuggggaacuguacaaccuucuagUuuucc.......................	1	1	Fm2
....uggggaacuguacaaccuucuagcuuucc.......................	13	0	Fm2
....uggggaacuguacaaccuucuagcGuucc.......................	1	1	Fm2
....ggggaacuguacaaccuucuag.............................	8	0	Fm2
....cuguacaaccuucuagcu...........................	3	0	Fm2
....cuguacaaccuucuagcuuuc........................	29	0	Fm2
....cuguacaaccuucuagcuuucU.......................	2	1	Fm2
....cuguacaaccuucuagcGuucc.......................	9	1	Fm2
....cuguacaaccCucuagcuuucc.......................	2	1	Fm2
....cuguacaaccuucuagcCuucc.......................	8	1	Fm2
....cuguacUaccuucuagcuuucc.......................	1	1	Fm2
....Uuguacaaccuucuagcuuucc.......................	2	1	Fm2
....cuguacaaccuucuagcAuucc.......................	4	1	Fm2
....cuguacaaccuucuagGuuucc.......................	1	1	Fm2
....Nuguacaaccuucuagcuuucc.......................	3	1	Fm2
....cuguacaUccuucuagcuuucc.......................	1	1	Fm2
....cuguacaaccuCcuagcuuucc.......................	1	1	Fm2
....cuguacaaccAucuagcuuucc.......................	2	1	Fm2
....cAguacaaccuucuagcuuucc.......................	1	1	Fm2
....cuguacaaccuucuagcuuucc.......................	1128	0	Fm2
....cuguacaaccuucuagUuuucc.......................	4	1	Fm2
....cuguacaaccuucuagAuuucc.......................	8	1	Fm2
....cuguacaaccuucuagcuuuccA......................	27	1	Fm2
....cuguacaaccuucuagcuuuccU......................	29	1	Fm2
....uguacaaccuucuagcuuucc.......................	7	0	Fm2
..................ucugagguaguagguuguauagu...	10	0	Fm1
..................ucugagguaguagguugGauagu...	1	1	Fm1
..................ucugagguaguagguuguauaguu..	4	0	Fm1
...................cugagguaguagguuguauagu...	1	0	Fm1
...................Augagguaguagguuguauaguu..	1	1	Fm1
...................cugagguaguagguugAauaguu..	1	1	Fm1
...................cugagguaguagguuguauaguu..	6	0	Fm1
....................ugagguaguagguuguaA..	4	1	Fm1
....................ugagguaguaCguuguau..	2	1	Fm1
....................ugagguaguagguuguau..	95	0	Fm1
....................ugagguaguagguuguUu..	11	1	Fm1
....................ugUgguaguagguuguau..	1	1	Fm1

```
                    Mature                                       Star
caggagugggccccucagucugagguaguagguuguauaguuguguacuacaucaucuuggggaacuguacaaccuucuagcuuucccagagggcgccucaguacaacau
....................ugagguaguagguuguauU.......................................................................   131    1    Fm1
....................ugagguaguagguuguUua.......................................................................   2      1    Fm1
....................ugagguaguagguuguaAa.......................................................................   4      1    Fm1
....................ugagguaguagguuguaua.......................................................................   98     0    Fm1
....................ugagguaguagUuuguaua.......................................................................   1      1    Fm1
....................ugagguaguagguuguaCa.......................................................................   1      1    Fm1
....................ugagguaguagguuguUuag......................................................................   1      1    Fm1
....................ugagguaguagguuguauag......................................................................   643    0    Fm1
....................ugagguaguagguuguauaU......................................................................   3      1    Fm1
....................ugaAguaguagguuguauag......................................................................   1      1    Fm1
....................ugagguaguagguuguauUg......................................................................   1      1    Fm1
....................ugagguaguagguuguaCag......................................................................   3      1    Fm1
....................ugUgguaguagguuguauag......................................................................   2      1    Fm1
....................ugagguaguagguuguauaA......................................................................   7      1    Fm1
....................ugagguaguagguuguaAag......................................................................   3      1    Fm1
....................ugagguaguagguuguaGag......................................................................   5      1    Fm1
....................ugGgguaguagguuguauag......................................................................   1      1    Fm1
....................ugagguaguaCguuguauagu.....................................................................   9      1    Fm1
....................ugagguagCagguuguauagu.....................................................................   1      1    Fm1
....................ugagguaguaUguuguauagu.....................................................................   10     1    Fm1
....................ugaUguaguagguuguauagu.....................................................................   4      1    Fm1
....................Ggagguaguagguuguauagu.....................................................................   2      1    Fm1
....................ugagguaguagguuguauagC.....................................................................   4      1    Fm1
....................ugagguaguagguuguGuagu.....................................................................   12     1    Fm1
....................ugagguaguaAguuguauagu.....................................................................   23     1    Fm1
....................ugagAuaguagguuguauagu.....................................................................   1      1    Fm1
....................ugagguaguagguuguauagG.....................................................................   13     1    Fm1
....................uCagguaguagguuguauagu.....................................................................   1      1    Fm1
....................ugagguaguGgguuguauagu.....................................................................   3      1    Fm1
....................ugagguaguagguuguUuagu.....................................................................   10     1    Fm1
....................ugCgguaguagguuguauagu.....................................................................   10     1    Fm1
....................Agagguaguagguuguauagu.....................................................................   7      1    Fm1
....................ugagguaguagguuguauaCu.....................................................................   1      1    Fm1
....................uUagguaguagguuguauagu.....................................................................   1      1    Fm1
....................ugGgguaguagguuguauagu.....................................................................   13     1    Fm1
....................ugagguaguagAuuguauagu.....................................................................   1      1    Fm1
....................ugagguagGagguuguauagu.....................................................................   2      1    Fm1
....................ugagguaguagguuguauagu.....................................................................   9531   0    Fm1
....................uAagguaguagguuguauagu.....................................................................   2      1    Fm1
....................ugagguaguagguuguCuagu.....................................................................   24     1    Fm1
....................ugagguaguCgguuguauagu.....................................................................   1      1    Fm1
....................ugagguaguagguugAauagu.....................................................................   1      1    Fm1
....................ugagguaguagguuguauagA.....................................................................   8      1    Fm1
....................ugagguaguagguuUuauagu.....................................................................   3      1    Fm1
....................ugagguCguagguuguauagu.....................................................................   1      1    Fm1
....................ugaggAaguagguuguauagu.....................................................................   1      1    Fm1
....................ugagguaguagguuguaAagu.....................................................................   28     1    Fm1
....................ugaAguaguagguuguauagu.....................................................................   1      1    Fm1
....................ugagguNguagguuguauagu.....................................................................   1      1    Fm1
....................ugagguGguagguuguauagu.....................................................................   10     1    Fm1
....................ugagguaguagguuguauUgu.....................................................................   2      1    Fm1
....................ugagguaguagguuguaGagu.....................................................................   65     1    Fm1
....................ugagguaguaggCuguauagu.....................................................................   1      1    Fm1
....................ugagguaguagguuguaCagu.....................................................................   74     1    Fm1
....................ugagguaguagCuuguauagu.....................................................................   1      1    Fm1
....................ugUgguaguagguuguauagu.....................................................................   8      1    Fm1
....................Cgagguaguagguuguauagu.....................................................................   1      1    Fm1
....................ugagguaguagguGguauagu.....................................................................   1      1    Fm1
....................ugagguaguaggAuguauagu.....................................................................   1      1    Fm1
....................ugaggCaguagguuguauagu.....................................................................   2      1    Fm1
....................ugagguaguagguuguauGgu.....................................................................   1      1    Fm1
....................ugagguagAagguuguauagu.....................................................................   3      1    Fm1
....................Ngagguaguagguuguauagu.....................................................................   8      1    Fm1
....................ugagguaguagguuguaAaguu....................................................................   106    1    Fm1
....................ugaggGaguagguuguauaguu....................................................................   2      1    Fm1
....................ugGgguaguagguuguauaguu....................................................................   37     1    Fm1
....................ugagguagGagguuguauaguu....................................................................   4      1    Fm1
....................ugagguCguagguuguauaguu....................................................................   3      1    Fm1
....................Agagguaguagguuguauaguu....................................................................   8      1    Fm1
....................ugagguaguagguuguauagGu....................................................................   1      1    Fm1
```

Mature (ugagguaguagguuguauaguu) — **Star** (cuguacaaccuucuagcuuucc)

```
caggagugggccccucagucugagguaguagguuguauaguuguguacuacaucaucuuggggaacuguacaaccuucuagcuuucccagagggcgccucaguacaacau
....................ugagguaguagguuguauaguG....................................................................  25     1  Fm1
....................ugagguaguagguuguauaguC....................................................................  11     1  Fm1
....................ugaAguaguagguuguauaguu....................................................................  3      1  Fm1
....................ugagguaguagguuguauGguu....................................................................  1      1  Fm1
....................ugagUuaguagguuguauaguu....................................................................  2      1  Fm1
....................ugagguaguaUguuguauaguu....................................................................  21     1  Fm1
....................ugagguaguagguCguauaguu....................................................................  6      1  Fm1
....................ugagguGguagguuguauaguu....................................................................  13     1  Fm1
....................ugagguaguagguuguGuaguu....................................................................  35     1  Fm1
....................ugagguaguCgguuguauaguu....................................................................  2      1  Fm1
....................ugaCguaguagguuguauaguu....................................................................  1      1  Fm1
....................ugagguaguagguuguaCaguu....................................................................  159    1  Fm1
....................ugaggAaguagguuguauaguu....................................................................  6      1  Fm1
....................ugagguaguagguuguauUguu....................................................................  9      1  Fm1
....................ugagguaguagguAguauaguu....................................................................  6      1  Fm1
....................Cgagguaguagguuguauaguu....................................................................  16     1  Fm1
....................Ggagguaguagguuguauaguu....................................................................  4      1  Fm1
....................uCagguaguagguuguauaguu....................................................................  2      1  Fm1
....................ugagguaguagguuguauaguA....................................................................  131    1  Fm1
....................ugagguaguagguuguauagAu....................................................................  3      1  Fm1
....................ugagguaCuagguuguauaguu....................................................................  2      1  Fm1
....................ugagguaguagCuuguauaguu....................................................................  1      1  Fm1
....................ugCgguaguagguuguauaguu....................................................................  21     1  Fm1
....................Ngagguaguagguuguauaguu....................................................................  20     1  Fm1
....................ugagCuaguagguuguauaguu....................................................................  1      1  Fm1
....................ugagguaguagguuAuauaguu....................................................................  3      1  Fm1
....................ugagguaguagguuUuauaguu....................................................................  10     1  Fm1
....................ugagguaguagguugAauaguu....................................................................  3      1  Fm1
....................ugagguaguGgguuguauaguu....................................................................  25     1  Fm1
....................ugagguaguaggAuguauaguu....................................................................  2      1  Fm1
....................ugagAuaguagguuguauaguu....................................................................  1      1  Fm1
....................ugagguagCagguuguauaguu....................................................................  3      1  Fm1
....................ugaggCaguagguuguauaguu....................................................................  4      1  Fm1
....................ugagguaguUgguuguauaguu....................................................................  8      1  Fm1
....................ugagguUguagguuguauaguu....................................................................  6      1  Fm1
....................ugagguaguagguuguCuaguu....................................................................  67     1  Fm1
....................ugagguaguagguuguUuaguu....................................................................  28     1  Fm1
....................ugagguaguagguuguauagCu....................................................................  1      1  Fm1
....................ugagguaguaAguuguauaguu....................................................................  50     1  Fm1
....................ugagguagAagguuguauaguu....................................................................  1      1  Fm1
....................ugagguaguagguuguauaguu....................................................................  28805  0  Fm1
....................ugaggNaguagguuguauaguu....................................................................  4      1  Fm1
....................ugagguNguagguuguauaguu....................................................................  1      1  Fm1
....................ugUgguaguagguuguauaguu....................................................................  29     1  Fm1
....................uUagguaguagguuguauaguu....................................................................  8      1  Fm1
....................ugagguaguagguuguaGaguu....................................................................  155    1  Fm1
....................ugagguaguaggCuguauaguu....................................................................  8      1  Fm1
....................uAagguaguagguuguauaguu....................................................................  8      1  Fm1
....................ugagguaguagguuguauaUuu....................................................................  4      1  Fm1
....................ugagguaUuagguuguauaguu....................................................................  7      1  Fm1
....................ugagguaguagguugCauaguu....................................................................  3      1  Fm1
....................ugagguaguaCguuguauaguu....................................................................  32     1  Fm1
....................ugagguaguagguuguauaguuU...................................................................  2582   1  Fm1
....................ugagguaguagguuguauaguug...................................................................  33     0  Fm1
....................ugagguaguagguuguauaguuA...................................................................  382    1  Fm1
....................ugagguaguagguuguauaguuC...................................................................  73     1  Fm1
....................ugGgguaguagguuguauaguug...................................................................  1      1  Fm1
....................ugagguaguagguuguauaguuCu..................................................................  6      1  Fm1
....................ugagguaguagguuguauaguuUu..................................................................  132    1  Fm1
....................ugagguaguagguuguauaguuAu..................................................................  44     1  Fm1
....................ugagguaguagguuguauaguuguguacuacU..........................................................  1      1  Fm1
....................ugagguaguagguuguauaguuguguacuacaucaucuuU..................................................  2      1  Fm1
.....................gagguaguagguuguauagu.....................................................................  1      0  Fm1
.....................gagguaguCgguuguauaguu....................................................................  1      1  Fm1
.....................gagguaguagguuguauaguu....................................................................  12     0  Fm1
.....................Uagguaguagguuguauaguu....................................................................  1      1  Fm1
.....................gagguaguagguuguauaguuU...................................................................  1      1  Fm1
.............................................uacuacaucaucuuggggaacugua........................................  4      0  Fm1
.....................................................caucuuggggaacuguacaaccuucuagcuuuccU......................  1      1  Fm1
......................................................aucuuggggaacuguacaaccuucuagcuuucc.......................  2      0  Fm1
```

Mature **Star**

```
caggagugggccccucagucugagguaguagguuguauaguuguguacuacaucaucuuggggaacuguacaaccuucuagcuuucccagagggcgccucaguacaacau
.......................................................ucuuggggaacuguacaacc...................................	3	0	Fm1
.......................................................ucuuggggaacuguacaaccuucuagcuuucc.......................	3	0	Fm1
.......................................................ucuuggggaacuguacaaccuucuagcuuuccU......................	1	1	Fm1
........................................................cuuggggaacuguacaaccuucuagcu...........................	3	0	Fm1
........................................................cuuggggaacuguacaaccuucuagcuuucc.......................	3	0	Fm1
........................................................cuuggggaacuguacaaccuucuagcuuuccA......................	3	1	Fm1
........................................................cuuggggaacuguacaaccuucuagcuuuccU......................	2	1	Fm1
.........................................................uuggggaacuguacaacc...................................	1	0	Fm1
.........................................................uuggggaacuguacaaccuucu...............................	18	0	Fm1
.........................................................uuggggaacuguacaaccuucuagcuuucc.......................	20	0	Fm1
.........................................................uuggggaacuguacaacUuucuagcuuucc.......................	1	1	Fm1
.........................................................uuAgggaacuguacaaccuucuagcuuucc.......................	1	1	Fm1
.........................................................uuggggaacuguacaaccuucuagcuuuccU......................	6	1	Fm1
..........................................................uggggaacuguacaaccuucuagcuuucU.......................	1	1	Fm1
..........................................................uggggaacuguacaaccuucuagcuuucc.......................	12	0	Fm1
..........................................................uggggaacuguacaaccuucuagcuuuccA......................	1	1	Fm1
..........................................................uggggaacuguacaaccuucuagcuuuccU......................	1	1	Fm1
...........................................................ggggaacuguacaaccuucuag.............................	9	0	Fm1
............................................................gggaacuguacaaccuucuagU............................	8	1	Fm1
.................................................................cuguacaaccuucuagcu...........................	2	0	Fm1
.................................................................cuguacaaccuucuagcuu..........................	2	0	Fm1
.................................................................cuguacaaccuucuagcuuu.........................	3	0	Fm1
.................................................................cuguacaaccuucuagcCuu.........................	1	1	Fm1
.................................................................cuguacaaccuucuagcuCuc........................	1	1	Fm1
.................................................................cuguacaaccuucuagcAuuc........................	1	1	Fm1
.................................................................cuguacaaccuucuagcuuuc........................	158	0	Fm1
.................................................................cugCacaaccuucuagcuuucc.......................	1	1	Fm1
.................................................................cuguacaaccuucuagUuuucc.......................	8	1	Fm1
.................................................................cuguacaaccuucuagcuuuUc.......................	1	1	Fm1
.................................................................Uuguacaaccuucuagcuuucc.......................	3	1	Fm1
.................................................................cuguacaaccuucuagcGuucc.......................	20	1	Fm1
.................................................................Auguacaaccuucuagcuuucc.......................	1	1	Fm1
.................................................................cuguacaaccuucAagcuuucc.......................	1	1	Fm1
.................................................................cuguacaaccuucuagcuuucU.......................	31	1	Fm1
.................................................................cuguacaaccuucuUgcuuucc.......................	1	1	Fm1
.................................................................cuguacaaUcuucuagcuuucc.......................	1	1	Fm1
.................................................................cuguacaaccuucuagAuuucc.......................	14	1	Fm1
.................................................................cuguacaaccuucuagcuuucA.......................	4	1	Fm1
.................................................................cuguacaaccuuUuagcuuucc.......................	3	1	Fm1
.................................................................cuUuacaaccuucuagcuuucc.......................	1	1	Fm1
.................................................................cuguacaacUuucuagcuuucc.......................	8	1	Fm1
.................................................................Nuguacaaccuucuagcuuucc.......................	1	1	Fm1
.................................................................cAguacaaccuucuagcuuucc.......................	2	1	Fm1
.................................................................cuguacaaccGucuagcuuucc.......................	7	1	Fm1
.................................................................cuguacaaccuAcuagcuuucc.......................	1	1	Fm1
.................................................................cuguacaaccuucuagcAuucc.......................	20	1	Fm1
.................................................................cuguGcaaccuucuagcuuucc.......................	1	1	Fm1
.................................................................cuguacaaccuucuagGuuucc.......................	4	1	Fm1
.................................................................cuguacaaccuucuagcuuucc.......................	3132	0	Fm1
.................................................................cCguacaaccuucuagcuuucc.......................	1	1	Fm1
.................................................................cuguacaaccuucuagcCuucc.......................	16	1	Fm1
.................................................................cuguacaaccAucuagcuuucc.......................	3	1	Fm1
.................................................................cuguacaaccuucuagcuuuccU......................	78	1	Fm1
.................................................................cuguacaaccuucuagcuuuccA......................	168	1	Fm1
.................................................................cuguacaaccuucuagcuuuccG......................	4	1	Fm1
..................................................................uguacaaccuucuagcuuucc.......................	22	0	Fm1
..................................................................uguacaaccuucuagcuuuccA......................	12	1	Fm1
..................................................................uguacaaccuucuagcuuuccU......................	6	1	Fm1
```
